# Influence of cellulose nanocrystal formulations on the properties of pregelatinized cornstarch and cornmint essential oil films

**DOI:** 10.1038/s41598-025-03318-8

**Published:** 2025-05-30

**Authors:** Laras Putri Wigati, Ata Aditya Wardana, Francis Ngwane Nkede, Meng Fanze, Mohammad Hamayoon Wardak, M. A. Reshaka Kavindi, Tran Thi Van, Xirui Yan, Fumina Tanaka, Fumihiko Tanaka

**Affiliations:** 1 Faculty of Agriculture, Kyushu University, 744, Motooka, Nishi-ku, Fukuoka-shi, Fukuoka, 819-0395 Japan; 2 International Research Fellow of Japan Society for the Promotion of Science (JSPS), Postdoctoral Fellowship for Research in Japan (Standard), Tokyo, Japan; 3 Department of Food Technology, Faculty of Engineering, Bina Nusantara University, Jakarta, 11480 Indonesia; 4 Graduate School of Bioresource and Bioenvironmental Sciences, Kyushu University, 744, Motooka, Nishi-ku, Fukuoka-shi, Fukuoka, 819-0395 Japan; 5 Department of Research of Preservation Technology of Agricultural Products, Vietnam Institute of Agricultural Engineering and Postharvest Technology, Hanoi, 10000 Vietnam

**Keywords:** Biomaterial, Pregelatinized cornstarch, Edible coating, Nanocrystal, Colloids, Mechanical properties, Polymer characterization

## Abstract

More people are switching to biodegradable packaging, which is better for the environment and safer for food products. Meanwhile, edible films and coatings might be an option, they are easily degradable, safe for consumption, and environmentally friendly. This study aimed to evaluate the influence of various CNC formulations as Pickering emulsions with pregelatinized cornstarch incorporating essential oil of cornmint on the characterization of edible coating solutions and film. The development of edible films was using pregelatinized cornstarch (PCS), including cornmint essential oil (CEO) and cellulose nanocrystals (CNC). To examine the impact of the CNC content on the physicochemical, mechanical, and optical characteristics of these films, different concentrations (0.05, 0.07, 0.10, 0.25, 0.30, and 0.50% w/w) of CNC were added. The coating solution’s pH and viscosity, as well as the thickness and roughness of the film, usually rise with an increase in CNC concentration. Conversely, this led to a reduction in the weight loss during heating, elongation, and tensile strength, as well as Young’s modulus. The different amount of CNC (%) did not have a significant effect on the water solubility, hydrophobicity, color, water vapor permeability, or surface microstructure, as observed by atomic force microscopy. The addition of CNC to the film matrix reduced its mechanical strength but basically has low water vapor permeability and high surface hydrophobicity. Consequently, the films produced in this study can be effectively used as edible films in situations that demand strong resistance to water but do not require significant mechanical strength.

**Supplementary Information:**

The online version contains supplementary material available at 10.1038/s41598-025-03318-8.

## Introduction

The packaging industry stands at the forefront of addressing current environmental challenges, as unsustainable practices contribute significantly to global pollution and resource depletion. In response to heightened awareness and consumer demand for eco-friendly solutions, innovative packaging technologies have emerged, particularly through the development of biodegradable and edible coatings and films. These technologies represent a transformative shift in how food products are packaged, offering advantages that extend beyond mere convenience to encompass social, economic, and environmental benefits^1,2^.

Edible coatings and films are derived from natural substances and can be consumed along with the food products they protect. They serve multiple functions, including acting as barriers to moisture and gases, which helps extend shelf life and maintain food quality^3,4^. The adoption of these materials also aligns with the growing call for reducing plastic waste; traditional synthetic packaging contributes heavily to environmental degradation, whereas edible and biodegradable alternatives provide a means to minimize this impact^5,6^. Furthermore, the use of agricultural by-products as raw materials for these coatings and films not only reduces waste from the food industry but also promotes a circular economy by repurposing what would otherwise be discarded^3,7^.

As consumer preferences shift towards products that embody sustainability, the packaging industry is increasingly focusing on innovative solutions that incorporate biodegradable materials^8^. Moreover, incorporating natural antimicrobial agents into these coatings can further protect food from spoilage, presenting a dual benefit of prolonging shelf life while adhering to health standards^9,10^. The innovation in this sector not only responds to regulatory and societal pressures but also reinvents the concept of packaging from being purely protective to becoming an active participant in the preservation of food quality and safety^11,12^.

Polysaccharides are sustainable for fresh food and processed product packaging due to their optical and mechanical properties^13–15^. Due to its edible and biodegradable properties, starch is a popular polysaccharide alternative for plastics^14^. Starch is the main carbohydrate in biopolymer films and is abundant and sustainable. Due to their low cost, renewability, and natural breakdown, starch and associated byproducts are attractive edible film ingredients. Starch variability includes amylose content^16^. Starch is added to coatings to improve gelling and food adhesion^17^. Pregelatinized starch is made by briefly heating starch and drying it to powder. Edible coatings use it. Starch globules expand and dry to form a gel, gelatinizing at low temperatures. Pregelatinized starch is ideal for low-temperature edible coatings^18^.

Essential oils as natural antimicrobial agents are great for food preservation and food protection^19^. Food packaging materials like edible thin films and nano-emulsions are antimicrobial^20^. These oils flavor and preserve soft drinks, fizzy drinks, and seafood^21^. Mint family members include peppermint and cornmint. Steam-distilled plant leaves generate essential oils with up to 80% menthol. Commercial cornmint oils are usually fractionated and dementholized to remove most of the menthol. Both oils are acidic and sweet, thin, transparent, and scented. Both mint oils have comparable uses. They aid digestion and reduce headaches, muscle discomfort, colds, and sinusitis. They are popular oral hygiene products because they reduce body temperature and are antimicrobial. Cornmint oil is cheaper and more popular in fragrance and food manufacture. The pungent scent of these oils may detect plumbing leaks. Future market insights anticipate that aromatherapy interest worldwide will affect peppermint essential oil, which is more popular than cornmint. Natural insect repellents are popular because of Zika. Mint oil use in scent is projected to rise. India produces the most cornmint oil^22,23^, while North America produces most peppermint oil.

Solid-particle-stabilized Pickering emulsions are becoming more common. Pickering emulsion research is driven by culinary, cosmetic, and pharmaceutical needs. They are stable, biocompatible, easy to prepare, and consistent in size. Only solid particles prevent coagulation in Pickering emulsions. Acid hydrolysis-derived cellulose nanocrystals (CNC) Pickering stabilizers are promising. Cellulose nanocrystals (CNC) have better form, size, and surface quality control than cellulose nanofibrils in Pickering systems. The enhanced control makes emulsion droplet formation more consistent^24^.

The innovations made by the research, its novel discoveries, and its methods using smaller formulations of CNC for edible coatings and films production, as well as the combination of cornmint essential oil. The formulation and combination in this study have never been studied before. This study also examined how the formulation of Pickering emulsions (CNC) containing pregelatinized cornstarch and cornmint essential oil affected the characteristics of edible coating solutions and films. The coating solution properties were viscosity and pH, while the edible film properties were thickness, moisture content, water solubility, surface hydrophobicity, WVTR, WVP, thermal properties, color, transparency, elongation, tensile strength, Young’s modulus, and SEM and AFM microstructure.

## Results and discussion

### Viscosity

One important factor influencing the films’ thickness and microstructure is the coating solutions’ viscosity^25^. Viscosity has a correlation with microstructure in that the particle interactions within the solution. As indicated by Ulfa et al., a decrease in viscosity can reflect weakened intermolecular forces among the polymer constituents in the coating solution, which may allow for better film formation despite the risk of increased porosity^26^. Furthermore, studies have shown that a lower viscosity typically results in increased adhesion and uniformity of the coatings, which is crucial in applications involving food preservation^27,28^.

As shown in Table 1, the range of viscosities of the coating solutions is from 23.34 cP to 109.08 cP. The lowest solution viscosity was 0.05 at 23.34 cP, which gradually enhanced with the increase of CNC content, reaching a maximum of 109.08 cP (C0.50); when compared to the other treatment groups, this was significantly higher (*p* < 0.05). This could be explained by the CNC network structure forming in the polymer solution, which was constructed by strong hydrogen-bonding interactions, leading to an increase in viscosity^29^. A similar trend was observed in poly (ethylene oxide) solutions with different CNC contents, where the viscosity of the biocomposite solution gradually increased from 6 to 9 Pa.s (6000–9000 cP) after the addition of CNC 0, 5, 10, and 20 wt%, which was attributed to the formation of a CNC network structure^30^.

**Table 1:** Viscosity and pH of coating solutions with 0.05% Cellulose nanocrystal/CNC; 0.07% CNC; 0.10% CNC; 0.25% CNC; 0.30% CNC; and 0.50% CNC.

Films	Viscosity (cP)	pH
C0.05	23.34 ± 5.30^d^	2.62 ± 0.02^d^
C0.07	25.26 ± 5.72^ cd^	2.68 ± 0.02^bc^
C0.10	31.38 ± 1.52^ cd^	2.67 ± 0.02^c^
C0.25	33.78 ± 8.20^c^	2.68 ± 0.01^bc^
C0.30	57.16 ± 9.67^b^	2.71 ± 0.03^b^
C0.50	109.08 ± 8.01^a^	2.75 ± 0.01^a^

Results are expressed as mean ± standard deviation. Different letters indicate statistically significant differences at *p* < 0.05.

### pH

A film’s chemical composition and structure can be altered through adjusting its pH, which influences its mechanical properties, permeability, and roughness. For example, films fabricated at varying pH levels may differ in brittleness or elasticity^31^. The parameters of pH ranged from 2.62 to 2.75, with the treatment group’s mean value being 2.675 for coating solutions that contained CNC 0.07–0.25. As the CNC content increased from 0.25 to 0.50, the pH of the composite solution increased by 0.07 to reach a maximum value of 2.75. These results show that a higher amount of CNC significantly (*p* < 0.05) increased the pH solution. This may be attributed to the surface of CNC containing a large number of hydroxyl groups, which usually interact with water molecules and may release or interact with hydrogen ions (H^+^), and the solution’s pH is affected by this interaction, which raises the pH^32^. Furthermore, this increase was related to the subsequent mechanical properties (Table 1). Munir et al. conducted a study indicating that edible films produced under different pH conditions show notable changes in mechanical properties, particularly a reduction in tensile strength as pH levels increase^33^. The research demonstrated that changes in pH may undermine the structural integrity of the films by affecting molecular interactions, leading to a less cohesive film matrix. Espino-Díaz et al. emphasized that the mechanical properties of edible films are intricately connected to their chemical structure. pH variations influence polymer characteristics and the extent of cross-linking, which are essential for tensile strength and elongation^34^.

### Thickness

The thickness of the coating is crucial for determining the acceptability of the deposition, because it substantially influences the functionality of the coating. Specifically, thickness affects the permeability of the coating to water and gas, this consequently impacts the coated product’s biological stability and shelf life^35^. For edible coatings or films, a thickness of < 0.25 mm is ideal^36^. As shown in Table 2, the results of the interaction between the type of treatment and the concentration of CNC have a major impact on the edible coating’s thickness.

**Table 2:** Thickness and moisture content edible films with 0.05% Cellulose nanocrystal/CNC; 0.07% CNC; 0.10% CNC; 0.25% CNC; 0.30% CNC; and 0.50% CNC.

Films	Thickness (mm)	Moisture content (%)
C0.05	0.038 ± 0.003^c^	11.42 ± 0.69^c^
C0.07	0.043 ± 0.004^c^	11.93 ± 0.81^bc^
C0.10	0.044 ± 0.004^bc^	13.55 ± 0.34^a^
C0.25	0.046 ± 0.007^abc^	12.83 ± 0.41^abc^
C0.30	0.053 ± 0.005^ab^	12.78 ± 0.73^abc^
C0.50	0.054 ± 0.005^a^	12.91 ± 1.17^ab^

Results are expressed as mean ± standard deviation. Different letters indicate statistically significant differences at *p* < 0.05.

The findings of the variance analysis showed that the thickness of the edible coating was significantly increased with a *p*-value < 0.05 by the interaction between the CNC concentrations and treatment types. The thickness varied between 0.054 and 0.038 mm (Table 2), which is within the acceptable range for edible coatings (≤ 0.25 mm). The increase in thickness was proportional to the concentration of CNC because increasing the concentrations leads to a higher quantity of individual polymers and overall dissolved solids in the same volume^37^. The variation in thickness of edible coatings is determined by several factors, including the physical characteristics of the solution (such as drying time, viscosity, surface tension, and density) and the method used to produce the coating^38^. Furthermore, a thicker coating may restrict the interchange of respiration gases, leading to higher ethanol accumulation and increased off-flavors in the product^39^.

When the thickness of edible films increases, the internal network structure can become less pliable. Thicker films often result from a higher concentration of film-forming polymers in the solution or suspension. For instance, increased solid content in a chitosan solution leads to thicker films that exhibit decreased elongation at break and reduced elasticity, indicating a more rigid structure^40,41^. Studies have shown that thick films tend to have enhanced tensile strength, which implies that while they can withstand force without breaking, they are also less capable of deforming under stress, thereby demonstrating reduced flexibility^42,43^.

In addition to flexibility, the permeability of edible films to gases and moisture is significantly affected by their thickness. Thicker films generally exhibit lower water vapor permeability (WVP)^44,45^. This phenomenon can largely be attributed to the physical and structural attributes of the films. When the thickness of an edible film increases, it creates a more complex pathway for water vapor molecules to navigate, thereby enhancing the film’s overall barrier effectiveness against moisture transmission. Specifically, the increased thickness often leads to greater intermolecular interactions and a denser matrix within the film, which hinders the movement of water vapor molecules through it^46,47^.

### Moisture content

The water content of the edible coatings varied from 11.42 to 13.55% across the various combined treatments of CNC concentrations (Table 2). Moreover, maintaining low water levels is important, as this enhances the ability to reduce damage and prolong the food’s shelf life. The concentration of CNC significantly led to an increase in moisture content (*p*-value < 0.05), likely owing to the presence of more hydrophilic groups within the CNC^48^. However, when the CNC concentration exceeded 0.1%, the moisture content decreased. This could be attributed to the excess CNC causing a filling effect, hindering the contact between hydrophilic groups and water molecules, consequently reducing the porosity^49^.

The porosity of edible films affects their physical and mechanical properties, including moisture permeability, mechanical strength, and structural integrity. High porosity in edible films applied to food products can lead to a decline in quality and spoilage^50^ because the edible coating fails to function properly as a barrier. The use of materials such as chitosan can modify porosity to make it more compact, thereby reducing WVTR and enhancing protection against moisture loss^51,52^. Microstructure is closely related to porosity; when the polymer matrix is uneven, the film structure also becomes uneven^53^. Cross-linking agents can improve porosity, increase film density, and enhance mechanical properties. High porosity is correlated with low tensile strength and elasticity; cross-linking agents or plasticizers can help address these issues^54^.

### Water solubility

Water solubility is a key characteristic of films used in food packaging^55^. As shown in Table 3, the film solubility changed from 2.69% (C0.25) to 7.51% (C0.30) with increasing CNC content and increased slightly back to the initial value of 6.34% (C0.50), which may be due to the hydrogen bonding interactions between the pregelatinized cornstarch chains and the CNC hydroxyl groups in the film matrix, thus limiting the movement of low molecular weight polymers and other compounds in the water and improving the stability of the starch/cornmint essential oil (CEO)/CNC films^56^. However, excessive incorporation of CNC did not result in any significant changes (*p* > 0.05) in the water solubility. The research conducted by Yanti et al. and Golmohammadi et al. emphasizes the properties of CNC and microcrystalline cellulose (MCC) in film matrices^57,58^. While solubility was monitored, the observed variations did not indicate a significant effect of cellulose on solubility^57,58^. Nurhaliza et al. highlighted the advantageous properties of films incorporating both polysaccharides and bacterial cellulose, noting stable solubility ratios that demonstrate their efficacy as packaging materials while maintaining essential parameters^59^.

**Table 3:** Water solubility and contact angle of edible films with 0.05% Cellulose nanocrystal/CNC; 0.07% CNC; 0.10% CNC; 0.25% CNC; 0.30% CNC; and 0.50% CNC.

Films	Contact angle (θ)	Water solubility (%)
C0.05	110.32 ± 9.14^a^	6.00 ± 2.26^a^
C0.07	113.63 ± 7.76^a^	3.93 ± 1.30^a^
C0.10	100.49 ± 6.44^a^	5.07 ± 1.31^a^
C0.25	109.26 ± 15.02^a^	2.69 ± 2.22^a^
C0.30	114.86 ± 7.53^a^	7.51 ± 5.56^a^
C0.50	110.03 ± 2.54^a^	6.34 ± 2.43^a^

Results are expressed as mean ± standard deviation. Different letters indicate statistically significant differences at *p* < 0.05.

High solubility in edible films generally indicates greater susceptibility to degradation when exposed to moisture. For instance, films composed of cassava starch and soy protein concentrate have shown that higher solubility correlates with reduced water resistance, thereby increasing the risk of hydrolytic breakdown in high humidity scenarios^60,61^. The solubility levels reported for various edible films suggest that compositions with lower solubility values contribute to stronger barrier properties, which are essential for maintaining food quality during storage^62^. Notably, the physical structure of these films and the number of hydroxyl groups available for water interaction directly impact their moisture absorption capacity and influence their degradation rates under humid conditions^63^.

Meanwhile, the impact of relative humidity on the water vapor permeability indicates that with increasing humidity. For example, in a study on cassava starch-and-soy protein films, the water vapor permeability rises, leading to potential structural integrity issues^61^. Such films, when exposed to moisture, can absorb water and begin to hydrolyze, resulting in the breakdown of the film matrix and accelerated degradation and loss of functionality as a packaging material^61,64^. The solubility profile of these films also changes as they interact with water; films designed to dissolve in water for consumption will degrade more rapidly than those intended for protective packaging applications^65^.

### Surface hydrophobicity

**Fig. 1:**
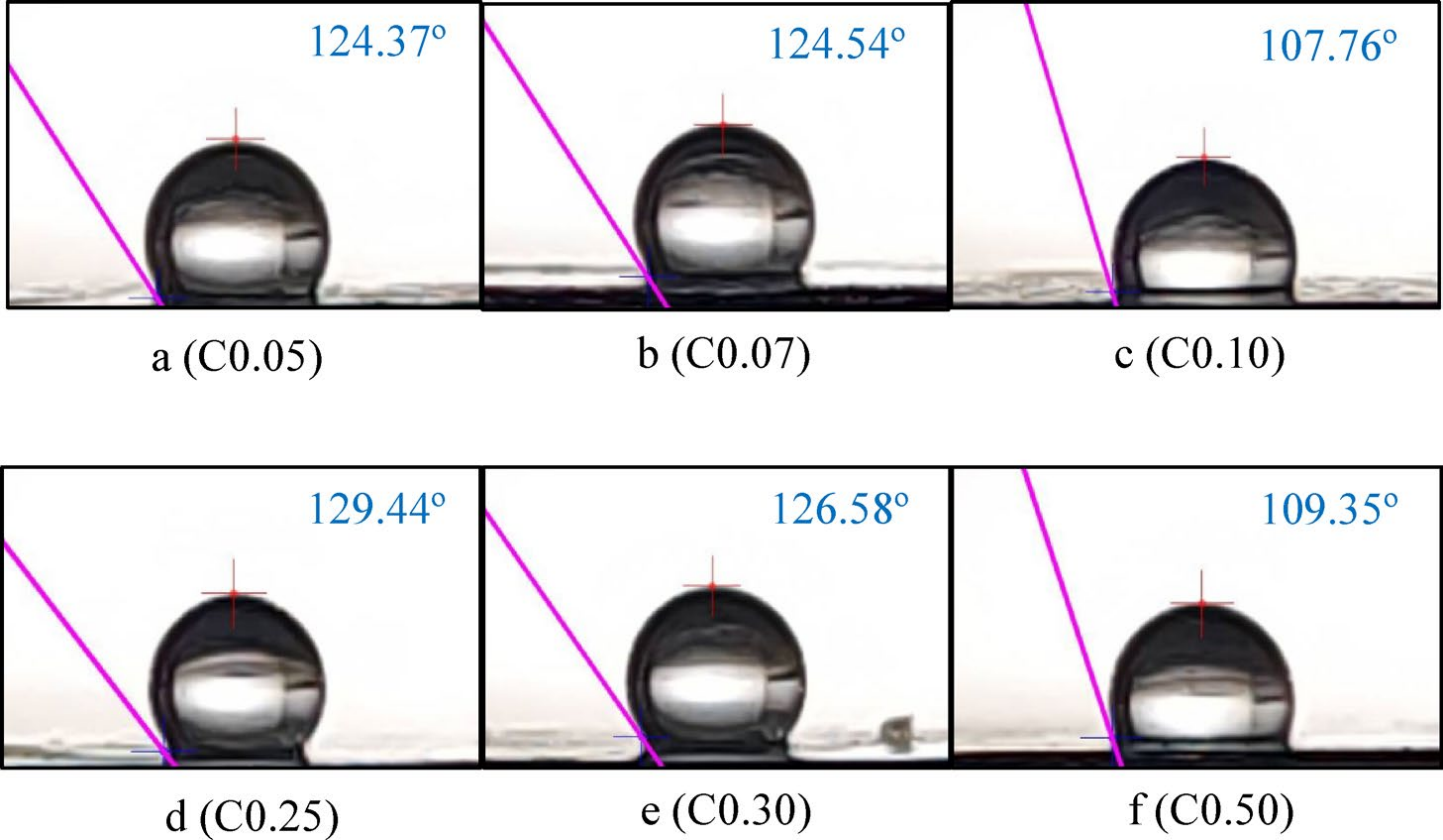
Contact angle appearances (highest values from each film). Edible film with 0.05% Cellulose nanocrystal/CNC (**a**); 0.07% CNC (**b**); 0.10% CNC (**c**); 0.25% CNC (**d**); 0.30% CNC (**e**); and 0.50% CNC (**f**).

Surface hydrophobicity, or WCA (water contact angle), is a crucial indicator for assessing the structural integrity of packaging materials in a moist or humid environment. A WCA value between 0° < θ < 90° indicates “hydrophilic,” while a value between 90° < θ < 150° indicate as a hydrophobic^66^. As shown in Fig. 1 and Table 3, the CNC-containing composite films were hydrophobic, reaching a maximum of 129.44° (C0.25). One possible reason may be the hydrophobicity of the CEO itself. In contrast, hydroxyl-rich cellulose chains can combine with water to form a hydrophobic barrier, thus increasing the film’s hydrophobicity. Similar results have been obtained in another study^67^. The WCA of the starch/CNC composite films also increased slightly as soon as the CNC content increased from 0 to 0.30%. However, the increase in hydrophobicity was not proportional to the amount of CNC added (*p* > 0.05), and the trend was similar to that observed for water solubility (Table 3).

### Water vapor transmission rate and water vapor permeability

The ability of a substance to transfer moisture to the surrounding environment or atmosphere is known as the water vapor transmission rate (WVTR). Moreover, Water Vapor Permeability (WVP) can help reveal the movement of mass and the way solutes interact with the functional characteristics of edible film^68^. The results for the WVTR and WVP in this investigation are listed in Table 4 indicates that no statistically significant distinction was observed (*p* > 0.05), and the inclusion of CEO and various CNC concentrations did not have a meaningful impact on WVTR and WVP (*p* > 0.05). Although the statistical significance was not clearly visible, the data reveal a noticeable trend as the usage of CNC increases; it still affects reduction in WVTR and WVP, yet the difference was statistically insignificant. This is possible because the difference between samples is not much and relatively the same.

**Table 4:** WVTR and WVP of edible films with 0.05% Cellulose nanocrystal/CNC; 0.07% CNC; 0.10% CNC; 0.25% CNC; 0.30% CNC; and 0.50% CNC.

Films	WVTR (g h^−1^ m^−2^)	WVP (g mm h^−1^ m^2^ kPa)
C0.05	0.00091 ± 0.00031^a^	0.00153 ± 0.00047^a^
C0.07	0.00078 ± 0.00005^a^	0.00123 ± 0.00018^a^
C0.10	0.00062 ± 0.00010^a^	0.00102 ± 0.00024^a^
C0.25	0.00061 ± 0.00009^a^	0.00097 ± 0.00013^a^
C0.30	0.00060 ± 0.00001^a^	0.00095 ± 0.00015^a^
C0.50	0.00058 ± 0.00015^a^	0.00127 ± 0.00053^a^

Results are expressed as mean ± standard deviation. Different letters indicate statistically significant differences at *p* < 0.05.

The decrease in the WVP suggests the formation of cohesive polymeric chains, indicating the presence of strong intermolecular interactions that hinder the passage of water vapor through the matrix. Starch, a hydrophilic substance, contains a significant number of hydrogen groups, the main cause of its insufficient water–vapor barrier qualities, is principally responsible. Nevertheless, acknowledging that edible starch films can serve as effective moisture barriers is important. To address this problem, it is recommended to use starches that have a high amylose and lipid content. The elevated amylose concentration in starch contributes to improved film formability, whereas lipids play a crucial role in regulating the barrier characteristics. The reduction in WVP, along with the higher CNC concentration, can be elucidated by the development of a labyrinthine structure when these particles are uniformly distributed in the polymer matrix. This structure hindered the diffusion of water vapor through the matrix. In addition, the presence of CNC can enhance the tortuosity of molecules of water inside the polymeric matrix, leading to a reduction in the WVP^69^.

### Thermal properties

Thermogravimetry (TGA) is performed to evaluate the degradation behavior of the composite films. Table 5 shows that all films exhibited three stages of weight loss. The first stage of weight loss below 143 °C is attributed to the process of moisture evaporation^70^ and this percentage is in line with the results of moisture content. The second stage below 362°C can be attributed to the depolymerization of CNC chains^71^ also the degradation of anhydro glucose rings of amylose and amylopectin of pregelatinized starch^72^. At this stage initial degradation temperature of composite films increased with increasing concentration of CNCs, as the highest was 143.4°C and the lowest was 112.2°C, observed for C0.05 and C0.07, respectively. However, the slight decrease at higher concentrations could be attributed to the agglomeration of CNCs, which reduced the integrity and crystallinity of the composite film, thus decreasing the degradation temperature^73^. In the third stage, the samples further disintegrate into carbonaceous residues^74^. At this stage, the lowest carbonaceous residues were observed for the C0.30, whereas the highest were observed for the C0.05 film. A previous study reported that the residual mass could be attributed to the incomplete combustion of organic substances, which was influenced by the inert atmosphere (N_2_) used during the measurement and the nature of the additives^75^. These results indicate the composite films exhibit excellent thermal stability up to 45 °C making them suitable for safe use in the food industry^76^.

**Table 5:** Temperature and weight loss related to thermogravimetry stages of edible films with 0.05% Cellulose nanocrystal/CNC; 0.07% CNC; 0.10% CNC; 0.25% CNC; 0.30% CNC; and 0.50% CNC.

Films	First stage		Second stage		Third stage		End	
	Temperature (°C)	Weight loss (%)	Temperature (°C)	Weight loss (%)	Temperature (°C)	Weight loss (%)	Temperature (°C)	Total weight loss (%)
C0.05	45–112.2	4.68	112.2–349.5	64.30	349.5–484.7	13.1	484.7	82.08
C0.07	45–143.4	5.95	143.4–349.1	64.52	349.1–483.0	12.49	483.0	83.82
C0.10	45 – 125.5	7.01	125.5–360.8	67.66	360.8–484.7	9.41	484.7	84.10
C0.25	45–135.8	6.97	135.8–358.2	69.45	358.2–484.6	8.68	484.6	85.10
C0.30	45–118.2	6.40	118.2–362.3	70.30	362.3 – 483.7	8.30	483.7	85.00
C0.50	45–116.8	6.80	116.8–349.4	66.31	349.4–485.3	11.04	485.3	84.15

### Color and transparency

Film color is an important factor that can influence consumer perceptions of food product^77^. Table 6 lists the various color parameters and transparency values of the prepared films. In this study, the CNC was the key factor affecting these parameters. The L* (lightness) value changed significantly with the addition of CNC (*p* < 0.05), whereas the a* (red to green) and b* (yellow to blue) values remained largely unchanged, and the CNC content didn’t significantly affect (*p* > 0.05) the a* and b* values (Table 6). The highest brightness (98.26) was observed for C0.05 films. The films with higher CNC concentrations exhibited reduced brightness. Similarly, another study found that adding 10% CNC to starch and grape pomace extracts reduced the brightness from 89.7 to 79.1^78^. Table 6 also shows that the films with 0.30% CNC (C0.30) exhibited increased redness (a*). The yellowness (b*) increased with the addition of CNC, and the highest intensity (5.09) was observed for the film containing 0.25% CNC (C0.25). This suggests that 0.25% CNC allows the cornmint essential oil to disperse more evenly^79^.

**Table 6:** Color L* (lightness), a* (redness), b* (yellowness) and transparency of edible films with 0.05% Cellulose nanocrystal/CNC; 0.07% CNC; 0.10% CNC; 0.25% CNC; 0.30% CNC; and 0.50% CNC.

Films	L*	a*	b*	Transparency
C0.05	98.26 ± 0.64^a^	0.23 ± 0.08^a^	4.90 ± 0.32^a^	10.85 ± 3.40^b^
C0.07	97.33 ± 0.56^c^	0.20 ± 0.09^a^	5.08 ± 0.25^a^	18.78 ± 2.28^a^
C0.10	97.97 ± 0.47^ab^	0.21 ± 0.11^a^	4.90 ± 0.40^a^	16.82 ± 3.87^ab^
C0.25	97.59 ± 0.39^bc^	0.23 ± 0.13^a^	5.09 ± 0.39^a^	15.30 ± 3.89^ab^
C0.30	97.57 ± 0.35^bc^	0.25 ± 0.15^a^	4.94 ± 0.39^a^	13.74 ± 3.18^ab^
C0.50	97.61 ± 0.29^bc^	0.23 ± 0.14^a^	4.98 ± 0.48^a^	12.18 ± 1.77^b^

Results are expressed as mean ± standard deviation. Different letters indicate statistically significant differences at *p* < 0.05.

Transparency in edible film plays a crucial role^80^ in both consumer perception and the functional performance of food packaging. The optical properties of these films significantly influence consumer acceptance, as transparency is a vital attribute for the visual assessment of food quality. Consumers generally prefer packaging that allows them to see the product inside, which can help them evaluate its freshness by the natural color and appeal^81–83^. Moreover, films that exhibit high transparency can imitate conventional polymeric packaging materials, thereby enhancing the aesthetic appeal of packaged products^81,84^. High transparency correlates with the ability of the film to allow adequate light transmission, which is critical for the preservation of food quality. Films with reduced transparency may hinder visual inspection and could potentially affect nutrient retention by blocking beneficial light^83^. For instance, studies have indicated that edible films with suitable optical properties can selectively filter UV radiation, thus protecting sensitive nutrients from degradation while still enabling consumers to view the product^85^.

**Fig. 2:**
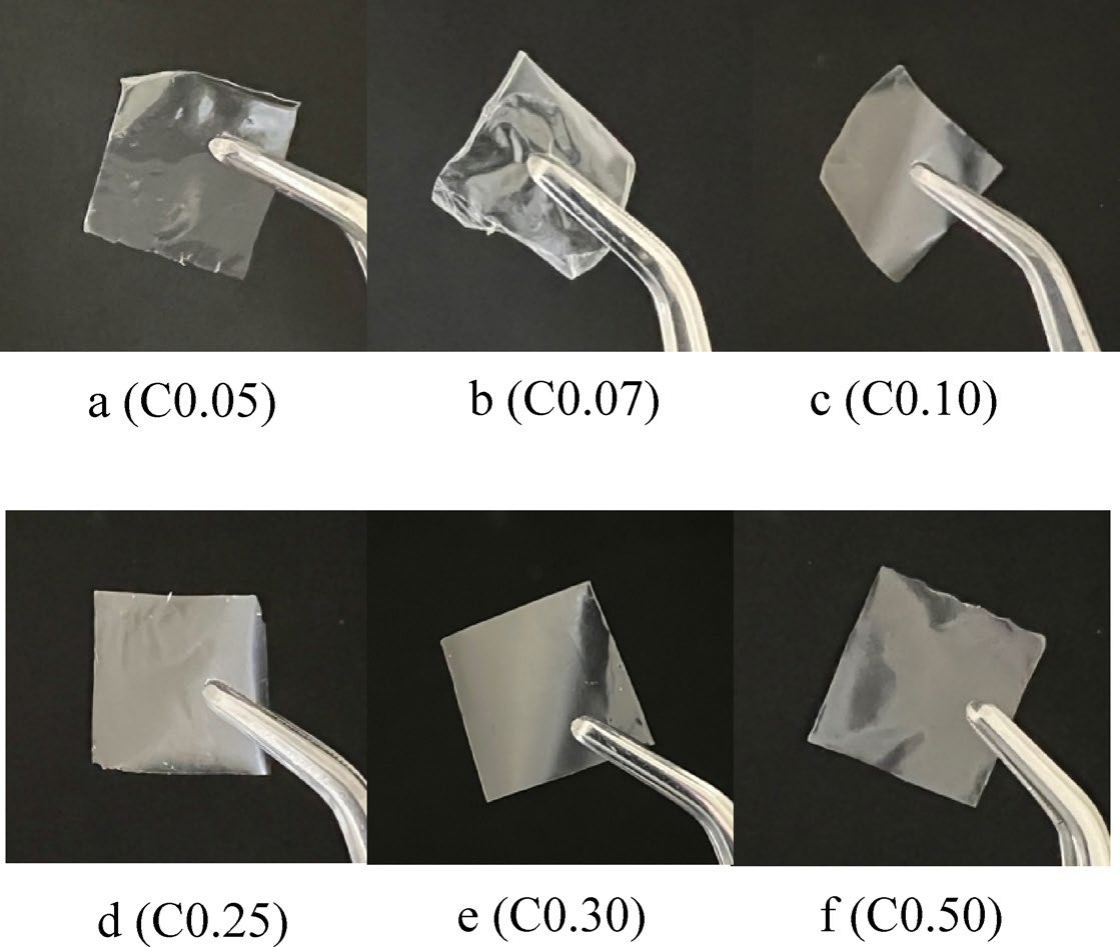
Colors and transparency appearance of edible films. Edible film with 0.05% Cellulose nanocrystal/CNC (**a**); 0.07% CNC (**b**); 0.10% CNC (**d**); 0.25% CNC (**d**); 0.30% CNC (**e**); and 0.50% CNC (**f**).

CNC significantly affected the transparency of the film, with the highest transparency value of 18.78, observed in C0.07 (0.07% CNC). Beyond this concentration, the transparency decreased as the CNC concentration increased, indicating greater opacity owing to light obstruction. Similar findings have also reported that the transparency of mung bean starch and ginger essential oil films decreases with the addition of tempo-oxidized cellulose nanocrystals^79^. Additionally, CNC reduces the transparency of carboxymethyl cellulose composite films^80^. Figure 2 illustrates the appearance of the film, which is consistent with the transparency results.

### Elongation, tensile strength, and Young’s modulus

The elongation analysis was performed in order to ascertain the longest length at which each edible film broke. When considering the packing materials made of film, this measurement is crucial. Table 7 displays the quantified data of elongation, tensile strength, and Young’s modulus. Statistical analysis revealed that the use of different CNC formulations resulted in substantial variations in the three mechanical parameters (*p* < 0.05). The flexibility of the film increases proportionally with elongation. Conversely, the formulation with the highest CNC concentration produced the lowest elongation of 2.81%, whereas the formulation with the lowest CNC concentration resulted in the highest elongation of 8.10%. Based on these results, it was discovered that a higher concentration of CNC in the edible coating solution led to a more rigid film. This indicates that the film had low elongation. Other investigations corroborated these findings, demonstrating that the elongation decreased as more CNC was added to the edible coating solution^86^. The elongation decreased because of the inflexible characteristics^55^. Table 7 demonstrates a significant reduction in the elongation at break as the CNC level increases. This can be explained by the presence of sufficiently high concentrations of CNC, which provided a strong and consistent network of reinforcements connected by hydrogen bonds. This structure restricts the stretching of chains^87^.

**Table 7:** Tensile strength, Young’s modulus and elongation edible films with 0.05% Cellulose nanocrystal/CNC; 0.07% CNC; 0.10% CNC; 0.25% CNC; 0.30% CNC; and 0.50% CNC.

Films	Tensile strength (Mpa)	Young modulus (Mpa)	Elongation (%)
C0.05	62.21 ± 9.48^ab^	66.91 ± 7.93^ab^	8.10 ± 5.04^a^
C0.07	70.92 ± 25.13^ab^	76.15 ± 26.65^ab^	7.66 ± 3.78^ab^
C0.10	83.01 ± 18.23^a^	87.48 ± 19.82^a^	5.28 ± 1.32^ab^
C0.25	88.45 ± 25.93^a^	91.87 ± 28.58^a^	3.50 ± 1.79^ab^
C0.30	77.10 ± 15.38^ab^	78.94 ± 15.62^ab^	2.42 ± 0.80^b^
C0.50	41.61 ± 6.06^b^	42.79 ± 6.33^b^	2.81 ± 0.46^ab^

Results are expressed as mean ± standard deviation. Different letters indicate statistically significant differences at *p* < 0.05.

The tensile strength varied between 41.61 MPa (C0.50) and 88.45 MPa (C0.25), as indicated in Table 7. The statistical examination of this parameter determined that, despite the absence of a continuous trend, the formulation of CNC significantly affected the variability of the tensile strength values. These results indicate that the quantity of CNC may affect the variance in tensile strength. Increasing the quantity of CNC utilized appeared to result in a decrease in tensile strength^88^. The tensile strength of edible films can be affected by the type and quantity of plasticizer employed, as well as the film’s thickness^89^. Further investigation of the use of edible starch films created by combining potato starch with CNC showed an inverse relationship between the elongation value and tensile strength. Specifically, higher elongation values correspond to lower tensile strengths and vice versa^69^. This result was expected because the significant compressive force caused the measured sample to become brittle, leading to reduced elongation, fracture, and reduced strength^90^. In the current investigation, the elongation was comparatively low, but the tensile strength was very high.

The Young’s modulus values (Table 7) varied between 42.79 MPa (C0.50) to 91.87 MPa (C0.25). The Young’s modulus is defined as the initial slope of the stress–strain curve. The Young’s modulus, also referred to as the elastic modulus, is a crucial parameter for quantifying the stiffness of films. The Young’s modulus is a measure of the stiffness of an edible film. A higher Young’s modulus indicated a stiffer edible film. Conversely, a lower Young’s modulus implies that the edible film possesses elastic characteristics^91^. This study found that a coating solution containing cornmint essential oils and a modified formulation of cellulose nanocrystals affected the fluctuation in Young’s modulus. Similar results were observed in other related investigations^92^. Young’s modulus can be affected by the level of molecular contact activity, leading to the formation of agglomerations and increased mobility of the molecular chain, resulting in greater elasticity of the films^25^. Conversely, if the molecular activity is low, the films are stiffer. In this study, it was shown that coating solutions with a low pH (specifically, a pH of 2) may contribute to an increase in the tensile strength and Young’s modulus. In contrast, the coating solutions with a pH of approximately 11 exhibited favorable mechanical properties^93,94^. However, the material’s natural characteristics may determine the coating’s mechanical properties^95^. Furthermore, films that exhibited reduced elongation tended to exhibit higher Young’s modulus^96^.

The concentration of CNC in edible films is a critical factor that affects interfacial bonding and network formation. Increasing concentrations of CNC can enhance the interfacial bonding within the polymeric matrix by establishing hydrogen bonds between hydroxyl groups on CNC and the polymer backbone, leading to a more compact and uniform film structure. For instance, CNC increases the availability of hydroxyl, carbonyl, and carboxyl functional groups, promoting better interaction with the polymer matrix^97^. Additionally, the presence of CNC contributes to the films’ overall crystallinity, which is essential for reducing water vapor permeability (WVP)^98,99^.

### Scanning electron microscope

**Fig. 3:**
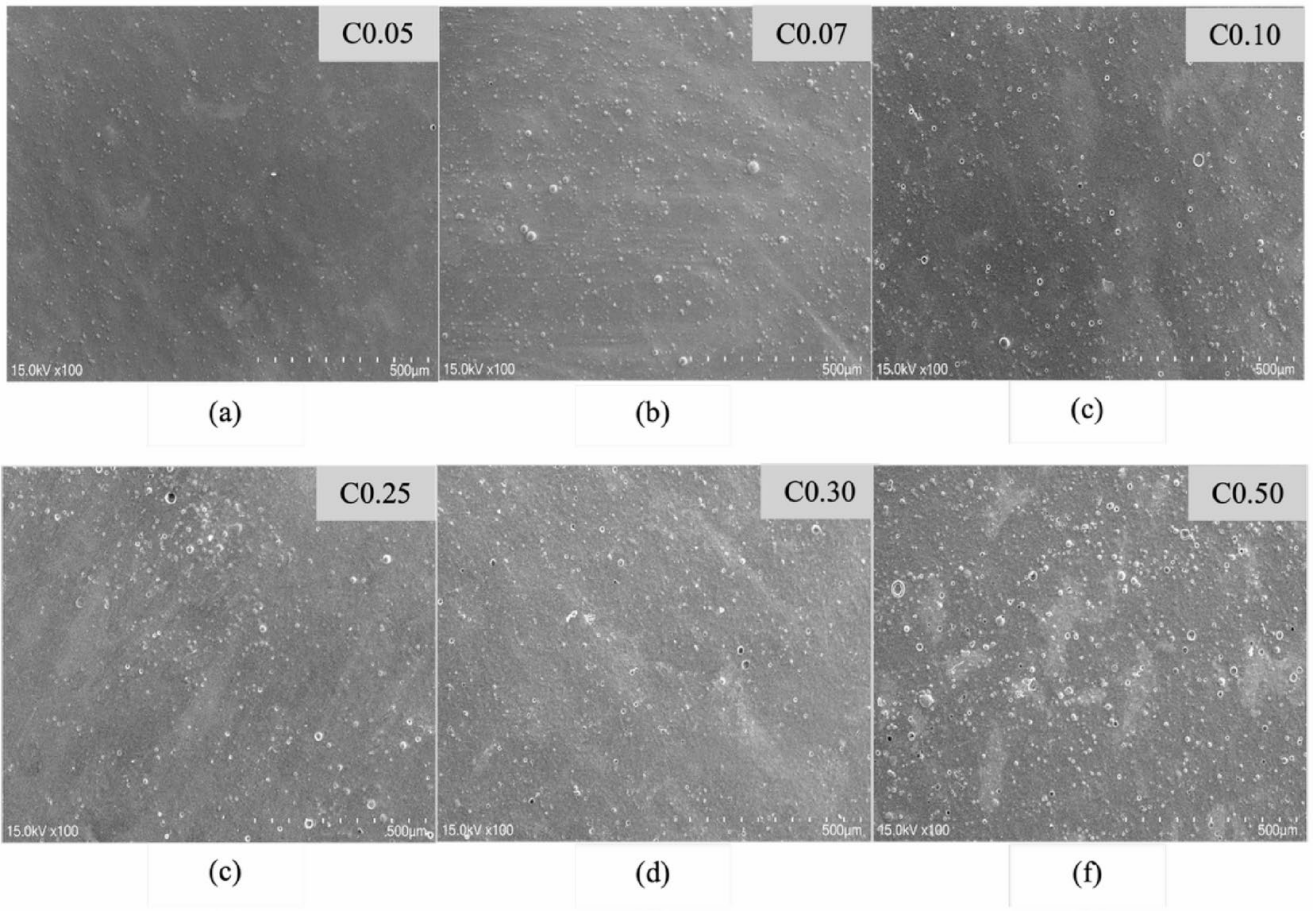
Scanning Electron Microscope (SEM) images of edible film surface. Edible film with 0.05% Cellulose nanocrystal/CNC (**a**); 0.07% CNC (**b**); 0.10% CNC (**d**); 0.25% CNC (**d**); 0.30% CNC (**e**); and 0.50% CNC (**f**).

SEM (scanning electron microscope) was used to analyze the microstructure of the edible films. Figure 3 shows the surface microstructures of the six films. Upon closer examination, it becomes noticeable that all six films exhibited gritty, uneven, and also rough textures. Additionally, many contained visible, insoluble CNC components that were heterogeneous along with oil droplets. The presence of nanocrystal aggregates in the film, as observed in previous studies on starch films incorporating CNCs, resulted in a rougher surface^100^.

As shown in Fig. 3, the films containing CNC 0.05 exhibited lower levels of CNC aggregates and had more regular and compact structures than the other films. This was likely due to the even distribution of droplets in the emulsion and the strong compatibility between the matrices. These features suggest that the emulsion remained stable during film preparation and drying, with no phase separation or droplet aggregation. The occurrence of Pickering emulsions may be attributed to the electrostatic stabilization of oil droplets through interactions between CNCs and EOs^70^. When the composite film structure is thick and has strong structural integrity, edible film properties should improve.

At higher amounts of CNC in the films, the surface microstructure exhibited fair heterogeneity and low compactness presence of some insoluble CNC particles, particularly at the highest CNC concentration of 0.50. The properties of oil droplets in a Pickering emulsion can affect their immobilization. Heterogeneity in edible films can affect mechanical strength, so variations in material distribution during film formation can influence mechanical properties such as tensile strength and elasticity^101,102^. Meanwhile, low compactness in edible films leads to high gas and moisture permeability, making this an important consideration when applying edible films to food products. Additionally, low crystallinity is typically associated with low film structural density, resulting in increased WVP^103^. In summary, the film microstructure findings confirmed the previously reported enhancements in characteristics resulting from the inclusion of CNCs and EOs^104^. The application of confessional cornstarch, which involves subjecting it to high temperatures to achieve gelatinization, as well as modified cornstarch, provides similar surface characteristics in edible film^70,105^.

### Atomic force microscope

**Fig. 4:**
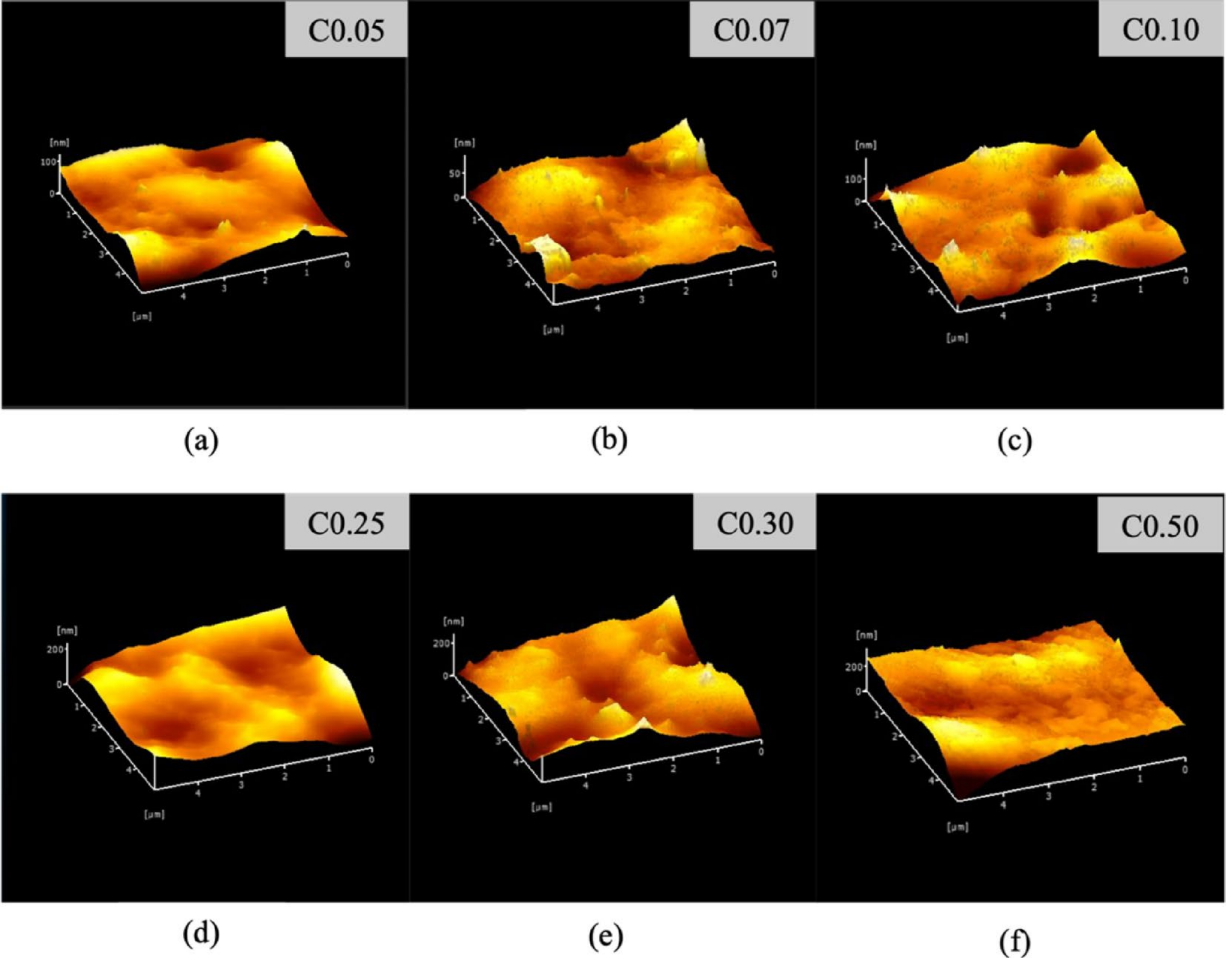
The three-dimensional morphology of Atomic Force Microscopy (AFM) images. Edible film with 0.05% Cellulose nanocrystal/CNC (**a**); 0.07% CNC (**b**); 0.10% CNC (**d**); 0.25% CNC (**d**); 0.30% CNC (**e**); and 0.50% CNC (**f**). Figures created by AFM software (Probe Station AFM5000II/Real TuneII, HITACHI, Japan).

AFM (atomic force microscopy) is a highly effective method for examining minor alterations in the surfaces of films resulting from the addition of filler materials and provides both qualitative and quantitative insights^106^. As seen in Fig. 4a, there was a tendency of fluctuating roughness with increasing levels of CNC compared with others (Fig. 4b–f), with average Ra and Rq values ranging from 4.14–2.05 to 6.84–2.35 nm, respectively (Table 8). No significant difference was found for Rq in all treatments; however, a significantly decreased roughness was observed for Ra at CNC 0.07% compared with CNC 0.25–0.50%-containing films. The lower smoothness was affected by the higher level of solid particle filler of the CNC inserted into the pregelatinized (PCS) biomatrix as a Pickering emulsion agent. The increased roughness of the PCS coating surface due to the addition of 0.25–0.50% CNC might be ascribed to the inhibition of CEO aggregation. The presence of CNC may maintain the size and distribution of the oil droplets. Moreover, these effects intensified during the evaporation phase of film formation^107^ when the water bound in the CNC was reduced. Consequently, the surface irregularities of the films were minimized. A previous study observed that the roughness of the film increased owing to the aggregation of oil within the gelatin-chitosan blend^108^. This phenomenon was visually confirmed by a 3D topography image, in which oil aggregates were formed, forming several spherical upland structures. In contrast, an earlier investigation documented that nanocellulose could clump together, leading to an increased roughness of the film owing to the creation of elevated areas on the surface^109^. This pattern aligns well with another study that noted the clustering of cellulose nanocrystals within the pectin matrix^110^ and chitosan^111^. A minor variation in the roughness properties implies that the study’s CNC concentration was optimal, maintaining the emulsifier’s fundamental function remained unaffected.

**Table 8:** Roughness characteristics of edible films with 0.05% Cellulose nanocrystal/CNC; 0.07% CNC; 0.10% CNC; 0.25% CNC; 0.30% CNC; and 0.50% CNC evaluated using Atomic Force Microscopy (AFM).

Films	Rq	Ra
C0.05	3.73 ± 2.49^a^	3.94 ± 2.76^ab^
C0.07	3.05 ± 3.01^a^	6.84 ± 2.74^a^
C0.10	2.05 ± 1.34^a^	5.00 ± 3.54^ab^
C0.25	2.85 ± 2.44^a^	2.35 ± 2.04^b^
C0.30	3.57 ± 0.61^a^	2.95 ± 0.64^b^
C0.50	4.14 ± 1.97^a^	2.75 ± 1.32^b^

Results are expressed as mean ± standard deviation. Different letters indicate statistically significant differences at *p* < 0.05.

Surface roughness contributes to the wettability of edible films. Smoother surfaces improve wettability, spreading film-forming liquids more evenly on food surfaces. Uneven surfaces weaken adhesion and performance, making this critical for coating application^112^. Optimization of surface qualities improves food substrate adhesion and film barrier properties^113,114^. Various biopolymer films with reduced roughness had lower contact angles, improving wettability and adhesion^115,116^. Physicochemical aspects of edible films, such as polymers and additives, affect adhesion. Surfactants and emulsifiers reduce surface tension, smoothen edible films, and improve substrate adhesion^117^. Roughness affects the mechanical characteristics of films, which affect their sealing ability, especially for moisture-rich meals where good sealing is essential to quality^118^. Adhesion, wettability, mechanical strength, and barrier characteristics are all affected by edible film structural integrity, which can be impaired by excessive roughness. Rougher surfaces may enhance gas and moisture permeability, reducing film protection^119^. Films with suitable texture qualities improve stability and mechanical capabilities, extending food shelf life^46,120^.

## Application on apples

### Color

**Fig. 5:**
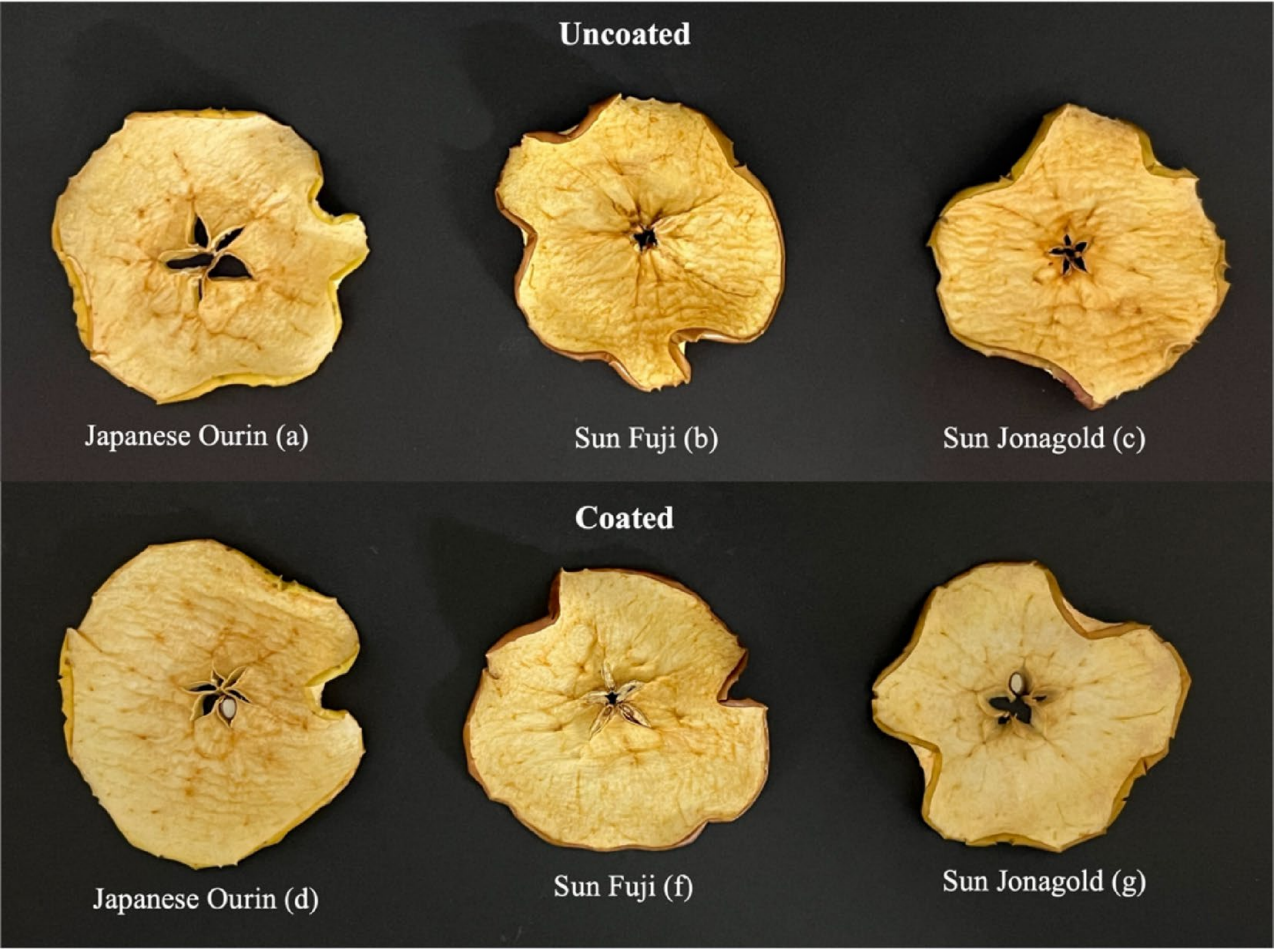
The color appearances of three different apple varieties, both uncoated and coated with 0.1% CNC.

Color including lightness is important parameter that determining visual quality and consumer acceptance. The color change of dried fruit slices can be influenced by the coating that was applied to the apple during the drying process. The average color parameters of lightness (L*), redness (a*), and yellowness (b*) for apples with and without coating for three different apple varieties (Sun Fuji, Sunjona Gold, and Ourin) are shown in Table 9. The appearance of dried apple slices with and without coating is shown in Fig. 5. When compared to apples without coating, dried apple slices of the Ourin variety (78.16) and Sun Fuji variety (75.88) can retain the lightness level when edible coating was applied, though the difference was not significant. The lightness level of the coated Sunjona Gold apple variety, however, was not higher than that of the uncoated apples (76.31 and 76.53). Coating could lessen the fruit’s surface’s exposure to oxygen, which would lessen oxidative browning as it dries. It is anticipated that coating fruit prior to drying will lessen oxidative damage^121^. Coating on fruit also might lower respiration and stop enzymes that responsible for enzymatic browning^122,123^. According to the study’s findings, an edible coating containing 0.1% CNC can act as an oxidation barrier. Some studies also found that polysaccharide-based coatings, including those enhanced with cellulose derivatives effectively reduce oxidative stress on fruits^122,124^. This is especially helpful during the drying process, where controlling moisture and oxygen levels can help to lower color changes and enhance fruit quality^125^.

**Table 9:** Color values of three different apple varieties, both uncoated and coated with 0.1% CNC.

Apples variety	Color					
	Uncoated			Coated (CNC 0.1%)		
	L*	a*	b*	L*	a*	b*
Japanese Ourin	76.68 ± 3.19^a^	7.22 ± 2.27^a^	30.48 ± 2.81^b^	78.16 ± 2.62^a^	6.46 ± 1.48^b^	31.87 ± 2.39^b^
Sun Fuji	72.30 ± 2.65^b^	8.11 ± 1.41^a^	34.86 ± 1.35^a^	75.88 ± 3.07^a^	8.70 ± 2.59^a^	33.42 ± 1.44^b^
Sunjona Gold	76.53 ± 1.44^a^	7.15 ± 2.12^a^	32.02 ± 2.85^b^	76.31 ± 3.11^a^	8.61 ± 1.27^ab^	36.74 ± 1.80^a^

Results are expressed as mean ± standard deviation. Different letters indicate statistically significant differences at *p* < 0.05.

## Conclusions

This study demonstrated that the addition of CNC to a PCS-based film matrix effectively maintained the water-barrier properties of the films. Notably, the addition of CNC to the edible coating solution led to a higher viscosity and pH, increased thickness, and increased weight loss during heating. The moisture content increased to 0.10 but then decreased to 0.50. The variation in the number of CNCs did not affect the surface hydrophobicity, water solubility, WVTR, or WVP, but it did have satisfactory results in terms of permeability. The variation in the CNC content also had no significant effect on the transparency of the film. Meanwhile, a higher CNC content degraded the mechanical properties of the films. On the surface of the film analyzed using SEM, it was found that a higher CNC content in the film will cause an uneven film surface, and there is no significant change in the film surface, as shown by the average roughness value of AFM. If viewed from the results of the analysis evenly, the more the use of CNC in making coating solutions will not make the film properties improve, thus the use of CNC around 0.05 to 0.10 is more recommended. However, additional research is required to evaluate the impact of these films on food applications.

Future studies will focus on the application of edible coatings for fruit drying processes and whether edible coatings in drying processes are able to maintain product quality. Further studies will also need to be conducted on varying the amount of CNC, followed by Fourier Transform Infrared Spectroscopy (FTIR) and X-Ray Diffraction Analysis (XRD) to analyze and calculate crystallinity, in order to understand how the increase in CNC affects the crystallinity of the films.

## Methods

### Materials

Edible coating solution, edible film: pregelatinized cornstarch (C*Hiform 12,748, Calgill, Japan), cornmint essential oil *Mentha arvensis* (un petite reve, Japan), cellulose nanocrystals with fiber dimensions 40–50 nm wide and 230–270 nm long, CAS Number: 9004-34-6 (Cellulose Lab, Canada), and propylene glycol (Wako Pure Chemical Industries, Ltd. Japan), citric acid (Wako Pure Chemical Industries, Ltd. Japan), distilled water produced from an automated water distillation device (Advantec® GS-200, Advantec® Tokyo, Japan). Apples (Japanese ourin, sun fuji, sunjona gold) were purchased at Gyomu Super (Itoshima, Fukuoka).

### Edible coating and film preparation

Two separate solutions were then prepared. The first solution consisted of pregelatinized cornstarch (2%), propylene glycol (1%), citric acid (1%), and distilled water. The second solution consisted of cellulose nanocrystals (0.05%, 0.07%, 0.10%, 0.25%, 0.30%, and 0.50%), cornmint essential oil (1%), and distilled water. Both solutions were individually homogenized using a rotor–stator homogenizer (T-25 digital ULTRA TURRAX®, IKA Japan Cooperation) at a speed of 15,000 rpm for 5 min each. Subsequently, the two solutions were combined and homogenized at the same speed for 5 min. A vacuum oven (ADP300; Yamato, Japan) was used to remove foam from the mixed solutions. The duration of the vacuuming procedure was 10 min. Subsequently, the film-casting process involved careful dispensing of 15 mL of each coating solution into a square silicon mold. The films were thereafter placed in a pre-set incubator (IN 802 Yamato, Japan) and subjected to a drying process at a temperature of 60 °C for a duration of 6 h. Once the films dried fully, they were removed from the silicon molds and placed in a desiccator containing a calcium chloride ${\text{(CaCl}}_{2}$) solution. This was performed to establish a consistent humidity level of 53% relative humidity (RH). The concentration of ${\text{CaCl}}_{2}$ used to prepare the solutions was 90%. Table 10 presents the full code and formulation details for each film.

**Table 10:** Edible coating formulations.

Sample	PCS (%)	CEO (%)	CNC (%)	PPG (%)	CAC (%)
C0.05	2	1	0.05	1.5	1.5
C0.07	2	1	0.07	1.5	1.5
C0.10	2	1	0.10	1.5	1.5
C0.25	2	1	0.25	1.5	1.5
C0.30	2	1	0.30	1.5	1.5
C0.50	2	1	0.50	1.5	1.5

*PCS* pregelatinized corn starch, *CEO* corn mint essential oil, *CNC* cellulose nanocrystal, *PPG* propylene glycol, *CAC* citric acid.

### Edible coatings solution analysis

#### Viscosity and pH

The viscosity of each solution in a 100 mL screw bottle was determined using a viscometer (DV2T Viscometer, BROOKFIELD AMETEK, USA) at a temperature of 20°C and a rotational speed of 10 rpm. The measurements were repeated ten times. Meanwhile, the pH measurement of each solution was conducted using a pH meter (COMPACT pH METER LAQUAtwin-pH-22, HORIBA Scientific, Japan) at a temperature of 20°C. The measurements were repeated five times^25^.

### Edible film analysis

#### Thickness and moisture content

The film thickness was measured using a ratchet thimble micrometer (Digimatic Micrometer, Model MDC-SX, Mitutoyo Corporation, Japan) at five specific sites for each film. Measurements were obtained in increments of 0.001 mm.

The moisture content was assessed by quantifying the weight reduction of the films. The films were divided into rectangular pieces of 3 cm × 3 cm using both scissors and a cutter. A stainless-steel dish devoid of any content was weighed using a reloading precision balance (FX-300i, A&D Company Limited, Australia). Subsequently, the cut sample was placed in a dish, and its weight was measured. The specimen was thereafter positioned in a constant-temperature drying oven (EO-300 V, ETTAS, AS ONE Corporation, Osaka, Japan) adjusted to a temperature of 105 °C for a duration of 24 h. Five samples were used to measure the moisture content for each treatment. The results were computed using Eq. (1) and is shown as a percentage (%)^25^.

1 $$Moisture \, \left( \% \right) \, = \frac{{(W_{1} - W_{2} )}}{{W_{1} }} \times 100$$

*W*_1_ is the initial weight of the sample before drying (g) and *W*_2_ is the weight of the sample after drying (g).

#### Water solubility

The water solubility of the films was determined using five replicates, with dimensions of 3 cm × 3 cm. The film’s initial dry weight (*Wf*) was calculated by subjecting it to a laboratory oven (WFO-520, EYELA Tokyo Rika Kikai Co. Ltd., Japan) at a temperature of 80°C for a duration of 48 h. Subsequently, each film sample was submerged in 25 mL of distilled water in an incubator (IN 802 Yamato, Japan) maintained at a temperature of 25 °C for a duration of 24 h. After 24 h of immersion, the remaining film pieces were filtered and weighed (*Wi*). They were then dried at 80 °C for 48 h until they reached a constant weight. The water solubility of the films was determined using Eq. (2)^25^.

2 $$W_{{\text{S}}} \left( \% \right) = \frac{{(W_{i} - W_{f} )}}{{W_{i} }} \times 100\%$$

#### Surface hydrophobicity

The surface hydrophobicity of the films was determined by measuring the contact angle using the Smart Contact Mobile Entry M411 device, developed by Exima Yokohama Lab Co., Ltd. in Japan. The 10 × 10 mm film sample was positioned on a metal specimen holder. Subsequently, a droplet of distilled water measuring 5 μL was applied to the surface of the film in five separate instances using a micropipette (Pipetmen, Gilson Inc. USA). The device was equipped with Smart Contact software, which was used to collect data and images of droplets^25^.

#### Water vapor transmission rate and water transmission rate

The water vapor permeabilities (WVP) of the films were determined in triplicate for each film type using the JIS Z 0208 technique. A round test cup with a diameter of 28 cm^2^ was employed to measure the water vapor permeability of the films. The film was then trimmed into a circular shape that exceeded the inner diameter of the cup. Anhydrous calcium chloride (15 g) was added to the cups as a desiccant at 0% relative humidity (RH). Subsequently, the edible film was placed on top of the permeable cup. Subsequently, the cup was enclosed in a lid and secured with a screw. The weight of the cup was measured before placing it in a temperature- and humidity-controlled chamber (TPAV-120-20, ISUZU CAP, Isuzu Seisakusho. Co., Ltd. Japan) set at a constant temperature of 25 °C and a relative humidity of 85%. The experiment utilized a MIDI LOGGER TYPE GL200A data logger manufactured by GRAPHTEC CORP. Japan monitors and records the temperature and humidity using its equipment. The weight of the cup was monitored every hour for 12 h. The formulas (Eqs. 3 and 4) were used to determine WVTR and WVP^25^.

3 $$WVTR = \left( {\Delta m/\Delta t} \right)A^{ - 1}$$

4 $$WVP = WVTR \times L\Delta p^{ - 1}$$

The symbol *∆m∕Δt* represents the rate at which moisture is gained in weight per unit of time, measured in grams per second (g s-1). The symbol A represents the surface area of the exposed film measured in square meters (m^2^). *L* represents the thickness of the film measured in millimeters (mm). The symbol *Δp* represents the difference in partial pressure.

#### Thermal properties

Thermogravimetry (TG) was employed to examine the weight reduction throughout the heating process, which was determined using a TG/DTA7300 instrument manufactured by Hitachi Hi-Tech Science Co., Ltd., Tokyo, Japan. An AI-type GAA-0068 (Hitachi Hi-Tech Science Co., Ltd. Tokyo, Japan). The measurement samples were adjusted to fit the size of the pan and weighed approximately 4 mg per sample. The samples were subjected to temperature measurements ranging from 45 to 500 °C, with a heating rate of 10 °C per minute. Atmospheric nitrogen was supplied at a flow rate of 20 mL per minute^25^.

#### Color

Color measurements were performed using a color reader, (CR-20; Konica Minolta Inc., Japan) against a white background. It was assumed that a fully transparent film would produce identical luminosity values (L*) to those obtained from the white calibration tile (L* = 100), and any deviation would indicate the presence of an opaque material (L* < 100). For this experiment, the film was positioned on a color reader and measurements were recorded at five distinct locations on each film^25^.

#### Transparency

The thin-film-coated samples were affixed to the cell side of a cuvette and analyzed using a UV–Vis spectrophotometer (Jasco, V-530, Japan) over the wavelength range of 200–800 nm. The data obtained were subsequently converted into transparency values. All measurements were performed in triplicate. The transmittance at 600 nm is denoted as T600, and the film thickness is represented by x (in mm)^126^.

5 $$Transparency = \frac{( - \log T600)}{x}$$

#### Elongation, tensile strength, and Young’s modulus

The films were divided into rectangular shapes measuring 1 × 5 cm to quantify the elongation. The films were secured using a motorized force test stand (FGS-50E-L; Nidec-Shimpo Co., Japan) with digital force gauges (FGPX-05; Nidec-Shimpo Co., Japan). The initial gap separation was established at 3 cm and then extended by displacing the grasp at a velocity of 60 mm s-1 until fracture occurred^25^. The percentage elongation was calculated using Eq. 6.

The tensile properties of the films were assessed by measuring their tensile strength, % elongation at break, and Young’s modulus. The tensile characteristics were determined using Eqs. 7 and 8.

6 $${\text{Percent elongation at breaking}} = \frac{{lf - l_{0} }}{{l_{0} }} \times 100$$

7 $${\text{Tensile strength}} = \frac{{F_{\max } }}{t \times w}$$

8 $${\text{Young}}^{\prime}s{\text{ modulus}} = \frac{Stress}{{Strain}} = \frac{F/A}{{\Delta l - l_{0} }}$$

where* l*₀ and *l*_f_ are represent the starting and final lengths of the gap, respectively.

F*max* represents the highest tensile force that can be applied,* t* is the thickness of the film, and *w* is the width of the sample.

Meanwhile, the relationship between the force applied to the structure (*F*), the cross-sectional area of the film (*A*), and the change in length of the film caused by the force (*Δl*).

#### Scanning electron microscope (SEM)

The film surface structure was analyzed using scanning electron microscopy (SEM) with an SU3500 model from Hitachi High-Technologies Corporation in Japan. The specimens were precisely shaped and affixed to conductive adhesive tape on both sides. The surface images were captured at a voltage of 15.0 kV and a magnification of 500 × ^25^.

#### Atomic force microscope (AFM)

Each edible film (10 mm × 10 mm) was placed on magnetic metal specimen discs for atomic force microscopy (AFM) (HITACHI AFM5000II, Hitachi High-Tech Corporation, Japan). The samples were scanned in non-contact mode using a sharpened cantilever of type SI-DF20. The scanning frequency was between 0.7 and 0.84 Hz, and the scanning area was 1 μm × 1 μm. The findings were also presented in the forms of *Ra*, which is an abbreviation for "roughness average," and *Rq*, which is an abbreviation for "root-mean-square roughness"^70,127^. *Rq* and *Ra* were measured in 9 separate replicates, and the mean values for each film were determined using Eqs. (9 and 10). Meanwhile, the included AFM software (Probe Station AFM5000II/Real TuneII) automatically created figures showing the three-dimensional morphology (3D) of AFM.

9 $$Rq = \sqrt {\frac{1}{n}\sum\limits_{i = 1}^{n} {Zi^{2} } }$$

10 $$Ra = \frac{1}{n}\sum\limits_{i = 1}^{n} {\left| {Zi} \right|}$$

### Application on apples

#### Coating and drying application

Three different apple varieties were used (Japanese ourin, sun fuji, and sunjona gold). Started with cleaning the apples with tap water, then distilled water. Left them to dry at room temperature, then continued with coating application (CNC 0.1%). After the apples completely dried, they were dipped in the coating solution for 1 min, then the coated apples dried at room temperature. For each variety, three apples were coated and then sliced to 3 mm thick using a mandoline adjustable slicer (Medove, China). For each apple, 10 slices were dried at 60 °C for 12 h (WFO-520, EYELA Tokyo Rika Kikai Co. Ltd., Japan). Dried uncoated apples were also used for control.

#### Color evaluation

The color of uncoated and coated apples was evaluated using a color reader (CR-20; Konica Minolta Inc., Japan). Each apple used three slices from different apples; each slice was then evaluated using a color reader three times with three different spots, and data averages were used. L* represents the lightness or darkness of dried apples, with 0 being black and 100 being white. Then a* indicates the redness or greenness of dried apples. Positive values represent redness, negative values represent greenness, and 0 represents neutrality. Furthermore, b* indicates the yellowness or blueness of dried apples. Positive values represent yellowness, negative values represent blueness, and 0 represents a neutral color.

### Statistical analysis

The effects of the treatments were assessed using analysis of variance (ANOVA), with the significance level set at *p* < 0.05. The experimental groups were compared using Tukey’s honest significant difference (HSD) test using IBM SPSS Statistics Version 29 (IBM SPSS Inc., USA).

## Electronic supplementary material

Below is the link to the electronic supplementary material.

Supplementary Material 1

## Data Availability

The datasets used and/or analysed during the current study are available in the supplementary file.
