# Supplementary material for: Influence of cellulose nanocrystal formulations on the properties of pregelatinized cornstarch and cornmint essential oil films
Source: Sci Rep. 2025 May 30;15:18997. doi: 10.1038/s41598-025-03318-8 (PMC12125276; doi:10.1038/s41598-025-03318-8)
Supplement: Supplementary file 1 — Supplementary Material 1 [file 41598_2025_3318_MOESM1_ESM.docx]

| Samples | Viscosity Coating solutions | | | | | |
| --- | --- | --- | --- | --- | --- | --- |
|  | C0.05 | C0.07 | C0.1 | C0.25 | C0.3 | C0.5 |
| 1 | 14.40 | 15.60 | 28.80 | 19.80 | 39.00 | 91.80 |
| 2 | 18.00 | 18.00 | 30.00 | 24.60 | 45.00 | 100.20 |
| 3 | 19.20 | 21.00 | 30.00 | 27.00 | 51.00 | 105.00 |
| 4 | 21.60 | 23.40 | 30.60 | 30.60 | 55.00 | 108.60 |
| 5 | 22.20 | 25.80 | 31.20 | 33.60 | 58.20 | 111.00 |
| 6 | 24.00 | 26.40 | 31.80 | 36.00 | 60.60 | 112.20 |
| 7 | 25.80 | 28.80 | 32.40 | 38.40 | 63.00 | 114.00 |
| 8 | 27.60 | 29.40 | 32.40 | 40.20 | 64.80 | 115.20 |
| 9 | 29.40 | 31.20 | 33.00 | 43.20 | 66.60 | 115.80 |
| 10 | 31.20 | 33.00 | 33.60 | 44.40 | 68.40 | 117.00 |
| AVERAGE | 23.34 | 25.26 | 31.38 | 33.78 | 57.16 | 109.08 |

**Table S1.** Raw data on the viscosity edible coating solutions with 0.05% Cellulose nanocrystal/CNC; 0.07% CNC; 0.10% CNC; 0.25% CNC; 0.30% CNC; and 0.50% CNC.

| Samples | pH | | | | | |
| --- | --- | --- | --- | --- | --- | --- |
|  | C0.05 | C0.07 | C0.1 | C0.25 | C0.3 | C0.5 |
| 1 | 2.65 | 2.70 | 2.70 | 2.68 | 2.72 | 2.76 |
| 2 | 2.62 | 2.66 | 2.68 | 2.67 | 2.75 | 2.75 |
| 3 | 2.60 | 2.68 | 2.66 | 2.67 | 2.71 | 2.77 |
| 4 | 2.63 | 2.67 | 2.66 | 2.67 | 2.70 | 2.74 |
| 5 | 2.62 | 2.69 | 2.67 | 2.69 | 2.68 | 2.75 |
| AVERAGE | 2.62 | 2.68 | 2.67 | 2.68 | 2.71 | 2.75 |

**Table S2.** Raw data on the pH edible coating solutions with 0.05% Cellulose nanocrystal/CNC; 0.07% CNC; 0.10% CNC; 0.25% CNC; 0.30% CNC; and 0.50% CNC.

| Samples | Thickness | | | | | | | | | | | |
| --- | --- | --- | --- | --- | --- | --- | --- | --- | --- | --- | --- | --- |
|  | C0.05 | | | Average | C0.07 | | | Average | C0.1 | | | Average |
|  | A | B | C |  | A | B | C |  | A | B | C |  |
| 1 | 0.037 | 0.040 | 0.039 | 0.039 | 0.052 | 0.051 | 0.045 | 0.049 | 0.043 | 0.054 | 0.048 | 0.048 |
| 2 | 0.033 | 0.039 | 0.033 | 0.035 | 0.043 | 0.040 | 0.036 | 0.040 | 0.042 | 0.040 | 0.038 | 0.040 |
| 3 | 0.048 | 0.037 | 0.044 | 0.043 | 0.044 | 0.041 | 0.036 | 0.040 | 0.040 | 0.042 | 0.043 | 0.042 |
| 4 | 0.032 | 0.037 | 0.035 | 0.035 | 0.047 | 0.042 | 0.043 | 0.044 | 0.039 | 0.044 | 0.041 | 0.041 |
| 5 | 0.042 | 0.034 | 0.040 | 0.039 | 0.043 | 0.046 | 0.039 | 0.043 | 0.046 | 0.055 | 0.046 | 0.049 |
|  |  |  |  | **0.038** |  |  |  | **0.043** |  |  |  | **0.044** |
|  |  |  |  |  |  |  |  |  |  |  |  |  |
|  |  |  |  |  |  |  |  |  |  |  |  |  |
| Samples | Thickness | | | | | | | | | | | |
|  | C0.25 | | | Average | C0.30 | | | Average | C0.50 | | | Average |
|  | A | B | C |  | A | B | C |  | A | B | C |  |
| 1 | 0.044 | 0.036 | 0.038 | 0.039 | 0.042 | 0.053 | 0.057 | 0.051 | 0.050 | 0.050 | 0.052 | 0.051 |
| 2 | 0.04 | 0.047 | 0.053 | 0.047 | 0.045 | 0.063 | 0.066 | 0.058 | 0.055 | 0.053 | 0.050 | 0.053 |
| 3 | 0.039 | 0.045 | 0.041 | 0.042 | 0.064 | 0.041 | 0.054 | 0.053 | 0.052 | 0.048 | 0.053 | 0.051 |
| 4 | 0.04 | 0.04 | 0.052 | 0.044 | 0.067 | 0.054 | 0.046 | 0.056 | 0.056 | 0.049 | 0.049 | 0.051 |
| 5 | 0.056 | 0.058 | 0.053 | 0.056 | 0.032 | 0.056 | 0.047 | 0.045 | 0.084 | 0.055 | 0.050 | 0.063 |
|  |  |  |  | **0.045** |  |  |  | **0.052** |  |  |  | **0.054** |

**Table S3.** Raw data on the thickness edible film with 0.05% Cellulose nanocrystal/CNC; 0.07% CNC; 0.10% CNC; 0.25% CNC; 0.30% CNC; and 0.50% CNC.

| Samples | Moisture content | | | | | |
| --- | --- | --- | --- | --- | --- | --- |
|  | C0.05 | C0.07 | C0.1 | C0.25 | C0.3 | C0.5 |
| 1 | 11.88 | 12.68 | 13.15 | 12.72 | 13.00 | 14.35 |
| 2 | 11.04 | 11.48 | 13.41 | 12.61 | 13.93 | 13.91 |
| 3 | 10.41 | 11.44 | 14.09 | 13.54 | 12.05 | 11.76 |
| 4 | 12.07 | 12.92 | 13.58 | 12.51 | 12.46 | 12.66 |
| 5 | 11.72 | 11.13 | 13.53 | 12.77 | 12.45 | 11.88 |
| AVERAGE | 11.43 | 11.93 | 13.55 | 12.83 | 12.78 | 12.91 |

**Table S4.** Raw data on the moisture content edible film with 0.05% Cellulose nanocrystal/CNC; 0.07% CNC; 0.10% CNC; 0.25% CNC; 0.30% CNC; and 0.50% CNC.

| Samples | Water solubility | | | | | |
| --- | --- | --- | --- | --- | --- | --- |
|  | C0.05 | C0.07 | C0.1 | C0.25 | C0.3 | C0.5 |
| 1 | 8.136 | 4.957 | 6.345 | 5.362 | 13.841 | 3.635 |
| 2 | 7.498 | 2.781 | 3.355 | 4.824 | 10.546 | 4.301 |
| 3 | 5.748 | 3.493 | 6.235 | 1.445 | 9.804 | 8.470 |
| 4 | 6.303 | 2.796 | 4.135 | 1.250 | 1.071 | 9.108 |
| 5 | 2.322 | 5.642 | 5.299 | 0.579 | 2.263 | 6.173 |
| AVERAGE | 6.001 | 3.934 | 5.074 | 2.692 | 7.505 | 6.337 |

**Table S5.** Raw data on the water solubility edible film with 0.05% Cellulose nanocrystal/CNC; 0.07% CNC; 0.10% CNC; 0.25% CNC; 0.30% CNC; and 0.50% CNC.

Surface hydrophobicity

| Samples | Contact angle | | | | | |
| --- | --- | --- | --- | --- | --- | --- |
|  | C0.05 | C0.07 | C0.10 | C0.25 | C0.30 | C0.50 |
| 1 | 107.70 | 110.95 | 100.73 | 129.44 | 126.58 | 107.45 |
| 2 | 124.37 | 124.54 | 98.04 | 114.12 | 115.01 | 109.35 |
| 3 | 100.88 | 103.72 | 104.82 | 114.16 | 114.57 | 108.36 |
| 4 | 104.90 | 111.74 | 107.76 | 94.39 | 112.44 | 111.12 |
| 5 | 113.73 | 117.20 | 91.09 | 94.21 | 105.72 | 113.86 |
| AVERAGE | 110.32 | 113.63 | 100.49 | 109.26 | 114.86 | 110.03 |

**Table S6.** Raw data on the contact angle edible film with 0.05% Cellulose nanocrystal/CNC; 0.07% CNC; 0.10% CNC; 0.25% CNC; 0.30% CNC; and 0.50% CNC.

| Sample | Rep | WVTR | Average WVTR | WVP | Average WVP |
| --- | --- | --- | --- | --- | --- |
| 0.05 | 1 | 0.001243 | 0.000907 | 0.002048 | 0.001529 |
|  | 2 | 0.000634 |  | 0.001148 |  |
|  | 3 | 0.000845 |  | 0.001392 |  |
| 0.07 | 1 | 0.000784 | 0.000777 | 0.001361 | 0.001229 |
|  | 2 | 0.000727 |  | 0.001024 |  |
|  | 3 | 0.000821 |  | 0.001300 |  |
| 0.10 | 1 | 0.000508 | 0.000615 | 0.000760 | 0.001019 |
|  | 2 | 0.000638 |  | 0.001059 |  |
|  | 3 | 0.000699 |  | 0.001239 |  |
| 0.25 | 1 | 0.000508 | 0.000607 | 0.000818 | 0.000966 |
|  | 2 | 0.000638 |  | 0.001019 |  |
|  | 3 | 0.000674 |  | 0.001060 |  |
| 0.30 | 1 | 0.000585 | 0.000597 | 0.001067 | 0.000952 |
|  | 2 | 0.000605 |  | 0.000777 |  |
|  | 3 | 0.000601 |  | 0.001013 |  |
| 0.50 | 1 | 0.000410 | 0.000584 | 0.000738 | 0.001274 |
|  | 2 | 0.000658 |  | 0.001291 |  |
|  | 3 | 0.000682 |  | 0.001794 |  |

**Table S7.** Raw data on the water vapor transmission rate (WVTR) and water vapor permeability (WVP) edible film with 0.05% Cellulose nanocrystal/CNC; 0.07% CNC; 0.10% CNC; 0.25% CNC; 0.30% CNC; and 0.50% CNC.

| Samples | | C0.05 | | | | | | | | | | Average | | |
| --- | --- | --- | --- | --- | --- | --- | --- | --- | --- | --- | --- | --- | --- | --- |
|  |  | L | a | b | L | a | b | L | a | b | L | | a | b |
| 1 | | 98.80 | 0.2 | 4.8 | 98.5 | 0.30 | 5.20 | 98.80 | 0.20 | 4.60 | 98.70 | | 0.23 | 4.87 |
| 2 | | 97.90 | 0.3 | 5.2 | 98.6 | 0.20 | 4.60 | 99.00 | 0.10 | 4.60 | 98.50 | | 0.20 | 4.80 |
| 3 | | 97.10 | 0.3 | 5.2 | 97.3 | 0.20 | 4.60 | 98.60 | 0.20 | 4.60 | 97.67 | | 0.23 | 4.80 |
| 4 | | 98.20 | 0.2 | 5.1 | 98.6 | 0.20 | 4.50 | 97.00 | 0.40 | 5.50 | 97.93 | | 0.27 | 5.03 |
| 5 | | 98.30 | 0.3 | 5.2 | 98.5 | 0.30 | 5.10 | 98.70 | 0.10 | 4.70 | 98.50 | | 0.23 | 5.00 |
| AVERAGE | | 98.06 | 0.26 | 5.10 | 98.30 | 0.24 | 4.80 | 98.42 | 0.20 | 4.80 | 98.26 | | 0.23 | 4.90 |
|  | |  |  |  |  |  |  |  |  |  |  | |  |  |
| Samples | | C0.07 | | | | | | | | | | Average | | |
|  |  | L | a | b | L | a | b | L | a | b | L | | a | b |
| 1 | | 97.20 | 0.1 | 4.9 | 97.4 | 0.20 | 4.90 | 97.70 | 0.20 | 5.40 | 97.43 | | 0.17 | 5.07 |
| 2 | | 96.60 | 0.4 | 5.4 | 98.5 | 0.20 | 5.10 | 98.20 | 0.20 | 5.30 | 97.77 | | 0.27 | 5.27 |
| 3 | | 97.40 | 0.2 | 5.2 | 96.4 | 0.40 | 5.60 | 97.20 | 0.10 | 4.80 | 97.00 | | 0.23 | 5.20 |
| 4 | | 96.80 | 0.2 | 5 | 97.7 | 0.00 | 4.70 | 97.30 | 0.10 | 5.00 | 97.27 | | 0.10 | 4.90 |
| 5 | | 97.10 | 0.1 | 4.9 | 97.5 | 0.20 | 5.00 | 96.90 | 0.20 | 5.00 | 97.17 | | 0.17 | 4.97 |
| AVERAGE | | 97.02 | 0.20 | 5.08 | 97.50 | 0.20 | 5.06 | 97.46 | 0.16 | 5.10 | 97.33 | | 0.19 | 5.08 |
|  | |  |  |  |  |  |  |  |  |  |  | |  |  |
| Samples | | C0.10 | | | | | | | | | | Average | | |
|  |  | L | a | b | L | a | b | L | a | b | L | | a | b |
| 1 | | 97.10 | 0.2 | 4.7 | 97.1 | 0.30 | 5.20 | 97.60 | 0.20 | 4.70 | 97.27 | | 0.23 | 4.87 |
| 2 | | 98.20 | 0.3 | 5.2 | 97.7 | 0.20 | 5.00 | 98.50 | 0.10 | 4.80 | 98.13 | | 0.20 | 5.00 |
| 3 | | 97.70 | 0.1 | 4.6 | 97.8 | 0.30 | 5.30 | 97.90 | 0.10 | 4.70 | 97.80 | | 0.17 | 4.87 |
| 4 | | 98.20 | 0.5 | 5.9 | 98.6 | 0.20 | 4.30 | 98.30 | 0.10 | 4.50 | 98.37 | | 0.27 | 4.90 |
| 5 | | 98.20 | 0.2 | 5.1 | 98.5 | 0.20 | 4.60 | 98.20 | 0.20 | 4.90 | 98.30 | | 0.20 | 4.87 |
| AVERAGE | | 97.88 | 0.26 | 5.10 | 97.94 | 0.24 | 4.88 | 98.10 | 0.14 | 4.72 | 97.97 | | 0.21 | 4.90 |
|  |  |  |  |  |  |  |  |  |  |  |  |  |  |  |
| Samples | | C0.25 | | | | | | | | | | Average | | |
|  |  | *L** | *a** | *b** | *L** | *a** | *b** | *L** | *a** | *b** | *L** | | *a** | *b** |
| 1 | | 97.70 | 0.2 | 5.1 | 98.1 | 0.20 | 5.30 | 97.50 | 0.10 | 4.80 | 97.77 | | 0.17 | 5.07 |
| 2 | | 96.80 | 0.6 | 5.9 | 97.6 | 0.30 | 5.20 | 97.70 | 0.20 | 4.90 | 97.37 | | 0.37 | 5.33 |
| 3 | | 97.10 | 0.3 | 5.1 | 97.6 | 0.20 | 4.80 | 97.70 | 0.10 | 4.60 | 97.47 | | 0.20 | 4.83 |
| 4 | | 97.00 | 0.4 | 5.8 | 98.3 | 0.20 | 4.80 | 97.80 | 0.10 | 4.80 | 97.70 | | 0.23 | 5.13 |
| 5 | | 97.50 | 0.3 | 5.4 | 97.5 | 0.10 | 4.70 | 97.90 | 0.20 | 5.20 | 97.63 | | 0.20 | 5.10 |
| AVERAGE | | 97.22 | 0.36 | 5.46 | 97.82 | 0.20 | 4.96 | 97.72 | 0.14 | 4.86 | 97.59 | | 0.23 | 5.09 |
|  | |  |  |  |  |  |  |  |  |  |  | |  |  |
| Samples | | C0.30 | | | | | | | | | | Average | | |
|  |  | *L** | *a** | *b** | *L** | *a** | *b** | *L** | *a** | *b** | *L** | | *a** | *b** |
| 1 | | 97.20 | 0.3 | 5 | 96.7 | 0.60 | 5.70 | 97.80 | 0.10 | 4.60 | 97.23 | | 0.33 | 5.10 |
| 2 | | 97.50 | 0.3 | 5 | 97.8 | 0.20 | 4.70 | 97.60 | 0.20 | 4.70 | 97.63 | | 0.23 | 4.80 |
| 3 | | 97.70 | 0.2 | 5 | 97.9 | 0.10 | 4.50 | 98.10 | 0.10 | 4.40 | 97.90 | | 0.13 | 4.63 |
| 4 | | 97.30 | 0.4 | 5.5 | 97.7 | 0.20 | 4.90 | 97.80 | 0.20 | 4.70 | 97.60 | | 0.27 | 5.03 |
| 5 | | 97.20 | 0.5 | 5.5 | 97.6 | 0.20 | 4.70 | 97.60 | 0.20 | 5.20 | 97.47 | | 0.30 | 5.13 |
| AVERAGE | | 97.38 | 0.34 | 5.20 | 97.54 | 0.26 | 4.90 | 97.78 | 0.16 | 4.72 | 97.57 | | 0.25 | 4.94 |
|  | |  |  |  |  |  |  |  |  |  |  | |  |  |
| Samples | | C0.50 | | | | | | | | | | Average | | |
|  |  | *L** | *a** | *b** | *L** | *a** | *b** | *L** | *a** | *b** | *L** | | *a** | *b** |
| 1 | | 97.40 | 0.4 | 5.5 | 97.7 | 0.10 | 4.70 | 97.50 | 0.20 | 5.00 | 97.53 | | 0.23 | 5.07 |
| 2 | | 97.20 | 0.6 | 6.2 | 97.5 | 0.20 | 4.80 | 97.60 | 0.10 | 4.80 | 97.43 | | 0.30 | 5.27 |
| 3 | | 97.60 | 0.2 | 4.9 | 97.8 | 0.20 | 4.40 | 97.80 | 0.10 | 4.80 | 97.73 | | 0.17 | 4.70 |
| 4 | | 97.90 | 0.2 | 4.7 | 96.9 | 0.40 | 5.40 | 97.90 | 0.20 | 4.60 | 97.57 | | 0.27 | 4.90 |
| 5 | | 97.60 | 0.2 | 5.6 | 97.9 | 0.20 | 4.60 | 97.90 | 0.10 | 4.70 | 97.80 | | 0.17 | 4.97 |
| AVERAGE | | 97.54 | 0.32 | 5.38 | 97.56 | 0.22 | 4.78 | 97.74 | 0.14 | 4.78 | 97.61 | | 0.23 | 4.98 |

**Table S8.** Raw data on the color edible film with 0.05% Cellulose nanocrystal/CNC; 0.07% CNC; 0.10% CNC; 0.25% CNC; 0.30% CNC; and 0.50% CNC.

Transparency

| Sample | C0.05 | | | C0.07 | | | C0.10 | | |
| --- | --- | --- | --- | --- | --- | --- | --- | --- | --- |
|  | T600 | Thickness | Transparency | T600 | Thickness | Transparency | T600 | Thickness | Transparency |
| 1 | 0.4999 | 0.039 | 7.788 | 0.1574 | 0.049 | 16.277 | 0.1948 | 0.048 | 14.698 |
| 2 | 0.3839 | 0.035 | 11.879 | 0.1996 | 0.040 | 17.643 | 0.1641 | 0.040 | 19.622 |
| 3 | 0.2114 | 0.043 | 15.695 | 0.1427 | 0.040 | 20.965 | 0.1365 | 0.042 | 20.757 |
| 4 | 0.3985 | 0.035 | 11.526 | 0.1683 | 0.044 | 17.589 | 0.1846 | 0.041 | 17.752 |
| 5 | 0.5181 | 0.039 | 7.386 | 0.1218 | 0.043 | 21.430 | 0.2811 | 0.049 | 11.248 |
| AVERAGE | 0.402 | 0.038 | 10.855 | 0.158 | 0.043 | 18.781 | 0.192 | 0.044 | 16.815 |
|  | | | | | | | | | |
| Sample | C0.25 | | | C0.30 | | | C0.50 | | |
|  | T600 | Thickness | Transparency | T600 | Thickness | Transparency | T600 | Thickness | Transparency |
| 1 | 0.1412 | 0.039 | 21.614 | 0.1225 | 0.051 | 17.997 | 0.1912 | 0.051 | 14.181 |
| 2 | 0.3069 | 0.047 | 10.993 | 0.2439 | 0.058 | 10.565 | 0.1823 | 0.053 | 14.036 |
| 3 | 0.2278 | 0.042 | 15.419 | 0.1703 | 0.053 | 14.505 | 0.2805 | 0.051 | 10.825 |
| 4 | 0.235 | 0.044 | 14.294 | 0.2572 | 0.056 | 10.594 | 0.2821 | 0.051 | 10.706 |
| 5 | 0.1623 | 0.056 | 14.186 | 0.2102 | 0.045 | 15.053 | 0.1989 | 0.063 | 11.133 |
| AVERAGE | 0.215 | 0.045 | 15.301 | 0.201 | 0.052 | 13.743 | 0.227 | 0.054 | 12.176 |

**Table S9.** Raw data on transparency edible film with 0.05% Cellulose nanocrystal/CNC; 0.07% CNC; 0.10% CNC; 0.25% CNC; 0.30% CNC; and 0.50% CNC.

| Samples | Elongatin (%) | | | | | |
| --- | --- | --- | --- | --- | --- | --- |
|  | C0.05 | C0.07 | C0.1 | C0.25 | C0.3 | C0.5 |
| 1 | 3.19 | 14.14 | 5.26 | 1.60 | 3.68 | 3.16 |
| 2 | 15.71 | 5.26 | 4.21 | 6.38 | 2.65 | 3.05 |
| 3 | 7.41 | 5.26 | 5.26 | 3.70 | 2.12 | 2.63 |
| 4 | 10.00 | 5.79 | 7.45 | 2.66 | 2.07 | 2.07 |
| 5 | 4.17 | 7.85 | 4.21 | 3.16 | 1.58 | 3.13 |
| AVERAGE | 8.09 | 7.66 | 5.28 | 3.50 | 2.42 | 2.81 |

**Table S10.** Raw data on elongation edible film with 0.05% Cellulose nanocrystal/CNC; 0.07% CNC; 0.10% CNC; 0.25% CNC; 0.30% CNC; and 0.50% CNC.

| Samples | Tensile strenght (Mpa) | | | | | |
| --- | --- | --- | --- | --- | --- | --- |
|  | C0.05 | C0.07 | C0.1 | C0.25 | C0.3 | C0.5 |
| 1 | 68.70 | 47.83 | 73.11 | 75.00 | 60.00 | 48.25 |
| 2 | 47.92 | 56.25 | 73.41 | 127.56 | 94.12 | 48.08 |
| 3 | 70.99 | 57.14 | 63.83 | 100.80 | 67.16 | 37.32 |
| 4 | 57.50 | 85.26 | 105.71 | 76.69 | 92.50 | 38.57 |
| 5 | 65.96 | 108.11 | 99.00 | 62.22 | 71.74 | 35.84 |
| AVERAGE | 62.21 | 70.92 | 83.01 | 88.45 | 77.10 | 41.61 |

**Table S11.** Raw data on tensile strenght edible film with 0.05% Cellulose nanocrystal/CNC; 0.07% CNC; 0.10% CNC; 0.25% CNC; 0.30% CNC; and 0.50% CNC.

| Samples | Young's modulus (Mpa) | | | | | |
| --- | --- | --- | --- | --- | --- | --- |
|  | C0.05 | C0.07 | C0.1 | C0.25 | C0.3 | C0.5 |
| 1 | 70.89 | 54.59 | 76.96 | 76.20 | 62.21 | 49.78 |
| 2 | 55.44 | 59.21 | 76.50 | 135.70 | 96.61 | 49.54 |
| 3 | 76.25 | 60.15 | 67.19 | 104.53 | 68.59 | 38.30 |
| 4 | 63.25 | 90.20 | 113.59 | 78.73 | 94.42 | 39.37 |
| 5 | 68.71 | 116.60 | 103.17 | 64.19 | 72.87 | 36.96 |
| AVERAGE | 66.91 | 76.15 | 87.48 | 91.87 | 78.94 | 42.79 |

**Table S12.** Raw data on Young's modulus edible film with 0.05% Cellulose nanocrystal/CNC; 0.07% CNC; 0.10% CNC; 0.25% CNC; 0.30% CNC; and 0.50% CNC.

|  |  |  |  |  |  |  |
| --- | --- | --- | --- | --- | --- | --- |
| Samples | AFM Ra | | | | | |
|  | C0.05 | C0.07 | C0.10 | C0.25 | C0.30 | C0.50 |
| 1 | 1.059 | 8.234 | 1.667 | 2.410 | 2.265 | 2.614 |
| 2 | 5.241 | 5.860 | 1.414 | 2.326 | 2.827 | 2.264 |
| 3 | 4.674 | 8.945 | 1.104 | 1.279 | 3.334 | 1.745 |
| 4 | 3.111 | 9.034 | 4.282 | 1.206 | 2.213 | 3.809 |
| 5 | 9.506 | 1.418 | 8.150 | 1.314 | 2.979 | 2.345 |
| 6 | 6.271 | 8.868 | 2.125 | 1.525 | 3.077 | 4.943 |
| 7 | 2.374 | 6.678 | 8.647 | 1.419 | 4.337 | 4.338 |
| 8 | 1.285 | 8.881 | 8.561 | 7.638 | 2.992 | 1.588 |
| 9 | 1.942 | 3.634 | 9.058 | 2.055 | 2.506 | 1.081 |
| AVERAGE | 3.940 | 6.839 | 5.001 | 2.352 | 2.948 | 2.747 |
|  |  |  |  |  |  |  |
|  |  |  |  |  |  |  |
| Samples | AFM Rq | | | | | |
|  | C0.05 | C0.07 | C0.10 | C0.25 | C0.30 | C0.50 |
| 1 | 1.362 | 1.077 | 2.037 | 3.231 | 3.030 | 4.153 |
| 2 | 6.405 | 7.445 | 1.789 | 2.787 | 3.628 | 3.846 |
| 3 | 5.186 | 1.027 | 1.493 | 1.573 | 3.896 | 2.543 |
| 4 | 4.394 | 1.109 | 5.029 | 1.511 | 3.013 | 6.132 |
| 5 | 1.115 | 1.827 | 1.200 | 1.583 | 3.481 | 4.400 |
| 6 | 8.222 | 1.035 | 3.477 | 1.722 | 3.568 | 7.305 |
| 7 | 3.017 | 8.590 | 1.152 | 1.706 | 4.921 | 5.562 |
| 8 | 1.614 | 1.111 | 1.137 | 9.154 | 3.602 | 1.948 |
| 9 | 2.279 | 4.246 | 1.154 | 2.391 | 2.951 | 1.394 |
| AVERAGE | 3.733 | 3.052 | 2.052 | 2.851 | 3.566 | 4.143 |

**Table S13.** Raw data on roughness characteristics (roughness average/Ra, and root-mean-square roughness/Rq) edible film with 0.05% Cellulose nanocrystal/CNC; 0.07% CNC; 0.10% CNC; 0.25% CNC; 0.30% CNC; and 0.50% CNC.

| Samples | Ourin | | | | | | | | | Average |
| --- | --- | --- | --- | --- | --- | --- | --- | --- | --- | --- |
|  | 1 | 2 | 3 | 1 | 2 | 3 | 1 | 2 | 3 |  |
| *L** | 70.10 | 74.2 | 76.2 | 77.1 | 77.20 | 77.40 | 79.80 | 81.30 | 76.90 | 76.69 |
| *a** | 11.8 | 8.8 | 9.3 | 6.6 | 6.50 | 5.90 | 5.20 | 4.90 | 6.00 | 7.22 |
| *b** | 34.1 | 32.9 | 34.9 | 29.1 | 29.60 | 28.50 | 27.40 | 27.50 | 30.40 | 30.49 |
|  |  |  |  |  |  |  |  |  |  |  |
| Samples | Sun Fuji | | | | | | | | | Average |
|  | 1 | 2 | 3 | 1 | 2 | 3 | 1 | 2 | 3 |  |
| *L** | 73.60 | 72 | 66.1 | 70.9 | 71.60 | 73.80 | 73.90 | 74.50 | 74.30 | 72.30 |
| *a** | 8.40 | 8.4 | 8.2 | 9.4 | 10.90 | 6.70 | 7.50 | 7.20 | 6.30 | 8.11 |
| *b** | 34.70 | 34.5 | 34.2 | 34.9 | 37.20 | 32.80 | 36.10 | 35.90 | 33.50 | 34.87 |

| Samples | Sun Jona Gold | | | | | | | | | Average |
| --- | --- | --- | --- | --- | --- | --- | --- | --- | --- | --- |
|  | 1 | 2 | 3 | 1 | 2 | 3 | 1 | 2 | 3 |  |
| *L** | 75.20 | 75.9 | 74.2 | 78.5 | 76.50 | 78.10 | 77.00 | 75.60 | 77.80 | 76.53 |
| *a** | 9.70 | 9.4 | 10.3 | 6 | 4.50 | 7.30 | 5.40 | 5.80 | 6.00 | 7.16 |
| *b** | 33.00 | 31.1 | 34.1 | 33.1 | 27.40 | 33.20 | 29.90 | 35.11 | 31.30 | 32.02 |

**Table S14.** Raw data on color characteristics values of uncoated three different apple varieties.

| Samples | Ourin | | | | | | | | | Average |
| --- | --- | --- | --- | --- | --- | --- | --- | --- | --- | --- |
|  | 1 | 2 | 3 | 1 | 2 | 3 | 1 | 2 | 3 |  |
| *L** | 79.6 | 79.7 | 76.2 | 81.6 | 78.5 | 80.4 | 73.4 | 75.5 | 78.6 | 78.17 |
| *a** | 5.6 | 5.6 | 8.8 | 5.2 | 6.0 | 5.0 | 8.6 | 7.7 | 5.7 | 6.47 |
| *b** | 31.4 | 32.5 | 34.7 | 27.7 | 31.3 | 29.3 | 34.7 | 34.0 | 31.3 | 31.88 |
|  |  |  |  |  |  |  |  |  |  |  |
| Samples | Sun Fuji | | | | | | | | | Average |
|  | 1 | 2 | 3 | 1 | 2 | 3 | 1 | 2 | 3 |  |
| *L** | 79.9 | 76.9 | 78.9 | 76.6 | 69.3 | 76.8 | 73.9 | 75.7 | 75.0 | 75.89 |
| *a** | 5.8 | 7.7 | 6.0 | 6.8 | 13.5 | 7.5 | 9.5 | 10.9 | 10.6 | 8.70 |
| *b** | 33.9 | 33.1 | 34.4 | 30.4 | 32.5 | 33.4 | 33.2 | 34.4 | 35.5 | 33.42 |

| Samples | Sun Jona Gold | | | | | | | | | Average |
| --- | --- | --- | --- | --- | --- | --- | --- | --- | --- | --- |
|  | 1 | 2 | 3 | 1 | 2 | 3 | 1 | 2 | 3 |  |
| *L** | 73.5 | 72.8 | 73.5 | 74.0 | 76.9 | 76.0 | 81.2 | 79.7 | 79.2 | 76.31 |
| *a** | 9.0 | 9.6 | 9.5 | 10.0 | 9.1 | 9.3 | 75.5 | 7.1 | 6.4 | 16.17 |
| *b** | 38.6 | 39.4 | 38.2 | 36.9 | 36.9 | 36.4 | 35.7 | 34.7 | 33.9 | 36.74 |

**Table S15.** Raw data on color characteristics values of three different apple varieties coated with 0.05% Cellulose nanocrystal/CNC 0.10%.
